# Resveratrol rescues cutaneous radiation-induced DNA damage via a novel AMPK/SIRT7/HMGB1 regulatory axis

**DOI:** 10.1038/s41419-022-05281-y

**Published:** 2023-01-01

**Authors:** Yi Jin, Xingyuan Liu, Xiaoting Liang, Jiabin Liu, Jieyu Liu, Zonglin Han, Qianxin Lu, Ke Wang, Bingyao Meng, Chunting Zhang, Minna Xu, Jian Guan, Li Ma, Liang Zhou

**Affiliations:** 1 Department of Toxicology, Guangdong Provincial Key Laboratory of Tropical Disease Research, School of Public Health, Southern Medical University, Guangzhou, China; 2 Department of Radiation Oncology, Nanfang Hospital, Southern Medical University, Guangzhou, China; 3 Guangdong Experimental High School, Guangzhou, China; 4 Institute of Molecular Immunology, School of Laboratory Medicine and Biotechnology, Southern Medical University, Guangzhou, China

**Keywords:** Translational research, Acetylation

## Abstract

Cutaneous radiation injury (CRI) interrupts the scheduled process of radiotherapy and even compromises the life quality of patients. However, the current clinical options for alleviating CRI are relatively limited. Resveratrol (RSV) has been shown to be a promising protective agent against CRI; yet the mechanisms of RSV enhancing radioresistance were not fully elucidated and limited its clinical application. In this study, we demonstrate RSV promotes cutaneous radioresistance mainly through SIRT7. During ionizing radiation (IR) treatment, RSV indirectly phosphorylates and activates SIRT7 through AMPK, which is critical for maintaining the genome stability of keratinocytes. Immunoprecipitation and mass spectrometry identified HMGB1 to be the key interacting partner of SIRT7 to mediate the radioprotective function of RSV. Mechanistic study elucidated that SIRT7 interacts with and deacetylates HMGB1 to redistribute it into nucleus and “switch on” its function for DNA damage repair. Our findings establish a novel AMPK/SIRT7/HMGB1 regulatory axis that mediates the radioprotective function of RSV to alleviate IR-induced cutaneous DNA injury, providing an efficiently-curative option for patients with CRI during radiotherapy.

## Introduction

Radiotherapy was originally designed to kill cancer cells or slow their growth by inducing DNA damage. However, the side effects of radiotherapy in normal tissues could not be ignored [1]. Nearly 85–95% of patients receiving radiotherapy developed different degrees of cutaneous radiation injury (CRI) [2] by showing erythema, dry and wet desquamation, secondary ulceration and cutaneous cancer, etc. [3], which seriously compromised the life quality of patients. The current clinical options using protective agents, including aloe vera gel, hyaluronic acid or vitamin C, etc., led to divergent therapeutic outcomes regarding effectiveness [4]. On the other hand, effective pharmaceuticals including triethanolamine cream and corticosteroids always accompanied with relative strong side effects during long-term application [5]. Therefore, mining effective therapeutic drugs for alleviating CRI is particularly important.

Resveratrol (Trans-3,5,4’-trihydroxystilbene, RSV) is a natural polyphenol plant antitoxin extracted from grapes, mulberry and polygonum cuspidatum [6]. RSV possesses a variety of biological functions including anti-tumor, anti-oxidative, anti-inflammatory and anti-aging activities [7–9], endorsing RSV to not only protect thymus, spleen, hippocampal, liver and intestinal from ionizing radiation (IR) [10–12], but also be an effective radioprotective agent for skin despite the lack of clear functional mechanism currently [13].

Human sirtuin family contains seven members of NAD^+^-dependent deacetylases with diverse roles in stress responses, metabolism modulation and cell senescence [14]. For example, SIRT1 takes part in inflammation and lipid metabolism by deacetylating different substrates in the cytoplasm and nucleus. SIRT2 also deacetylates a wide range of cytoplasmic and nuclear target proteins to influence microtubule dynamics, inflammation and differentiation. SIRT3, SIRT4 and SIRT5 are mostly localized and function in mitochondria [15]. SIRT6 and SIRT7 are nuclear-localized sirtuins with roles in maintaining genomic stability and transcription regulation [16]. Interestingly, accumulating evidence support that RSV is an effective sirtuin-activating compound (STAC) [17]. RSV has been considered to be the most effective SIRT1 activator [17] and plays multifaceted roles in obesity, diabetes, tumors and aging through allosteric activating of SIRT1 [18–20]. Also, RSV acts as a activator of SIRT3 and SIRT5 [21]. RSV activates SIRT3 to ameliorate cardiac fibrosis and improve cardiac function via the transforming growth factor-β/Smad3 pathway [22]. In short, RSV tightly relies on sirtuin family members to demonstrate its diverse functions.

In this study, to probe the downstream functional mediator of STAC pharmaceutical RSV in protecting skin from CRI, we screened the human sirtuins in keratinocytes and identified SIRT7 to be the highest-expressed nuclear sirtuin family member by analyzing transcriptomic sequencing data. Functional studies using in vitro and in vivo models revealed that RSV with defined concentration acts as a promising radioprotective agent to alleviate IR-induced DNA damage and cell death in keratinocytes and cutaneous tissues. Mechanistically, the radioprotective effect of RSV is dependent on AMPK-mediated SIRT7 phosphorylation and activation. Immunoprecipitation followed by high-performance liquid chromatography-mass spectrometry (HPLC-MS) identified HMGB1 to physically associate with and be deacetylated by SIRT7 which redistributes HMGB1 into nucleus and thus participates in DNA damage repair. The RSV-activated AMPK/SIRT7/HMGB1 regulatory axis identified in this study potentiates the application of RSV as a radioprotective agent and provides an efficiently-curative option for patients with CRI during radiotherapy.

## Materials and methods

### Cell lines

The human benign epidermal keratinocyte cell line HaCaT (Male, CellCook Biotech Co. Ltd.) were cultured in Dulbecco’s modified Eagle medium (Corning) supplemented with 10% fetal bovine serum (ExCell Bio, FSP500) and maintained at 37 °C with 5% CO_2_ in a humidified atmosphere. The STR profiles authentication information was listed in Supplementary Fig. S4. This cell line has been verified to be mycoplasma free.

### Animal studies and IR treatment

Male mice (BALB/C, 4–6 weeks old) for CRI study were purchased from the animal center of Southern Medical University. Mouse transportation, housing, and breeding were implemented in accordance with the recommendations of “The use of non-human animals in research”.

To explore the protective effect of RSV against IR, RSV was dissolved in physiological saline with 20% propylene glycol and 0.5% azone. The left and right posterior limb of the 6-week-old mice were depilated with depilatory cream, and then the solvent or RSV was applied to the skin of posterior limbs before and after the IR treatment as showed in Fig. 6a (each group *n* = 3). Mice left posterior limbs were exposed to 40 Gy X-ray after anesthesia using Elekta Precise medical linear accelerator (radiation type X-ray, Energy 6 MV, Dose Rate 300 MU/min). Each mouse was randomly assigned to each group and the investigator was blinded to the group allocation.

### RT-qPCR

Total RNAs from cells were extracted using TransZol reagent (TransGen Biotech Co., Ltd.) according to the manufacturer’s instructions. Reverse transcription was performed using TransScript Uni All-in-One First-Strand cDNA Synthesis SuperMix for qPCR (One-Step gDNA Removal) (TransGen Biotech, AU341). The mRNA expression levels were detected using Unique Aptamer Green Master Mix (Novogene, Tianjin, China) on LightCycler 96 Detection System (Roche, Basel, Switzerland). Detection of GAPDH mRNA expression was used for normalization. Primers used in this study are listed in Supplementary Table S1.

### DNA constructs

The SIRT7 and HMGB1-GFP expressing constructs for mammalian cell expression and SIRT7-GST construct for in vitro *E.coli* expression and following purification was purchased from YouBio Biological Technology Co., Ltd. (http://www.youbio.cn) with sequencing verifications.

### Western blot analysis

The antibodies used in this study are listed in Supplementary Table S2. Luminata Forte Western HRP substrate (Millipore) was used to detect the bound antibodies.

### Immunofluorescence staining

Immunofluorescence staining was performed as previously described [23]. Cells were fixed and then permeabilized with 0.5% NP-40 for 10 min, non-specific binding was blocked with 5% BSA in PBS for 1 h. Cells were then incubated with the primary antibody overnight at 4 °C, followed by incubation in dark with the secondary antibody for 1 h. Nuclei were stained with DAPI. Cells harboring γ-H2AX foci more than 15 were defined as positive cells. In this study, γ-H2AX (Santa Cruz Biotechnology, sc-517384, 1:250) was used as primary antibodies, Alexa Fluor® 488 (green, 1:250) was used as secondary antibodies.

### Coimmunoprecipitation (Co-IP) assay

Pierce crosslinking magnetic IP/Co-IP kit (Thermo Scientific) was used to perform IP/Co-IP assay. Control IgG or specific antigen antibodies (5 μg) was bound to protein A/G magnetic beads through 15-min incubation at room temperature. Disuccinimidyl suberate (20 μM) was used for crosslinking the bound antibody. Cells were lysed in precooled IP wash buffer and then centrifugated at 12,000 × *g* for 5 min. The supernatant was incubated with various specific antibodies or irrelevant IgG in the presence of protein A/G magnetic beads for 1 h, which was on a rotator and at room temperature. After stringent washing, the proteins were eluted from the magnetic beads using elution buffer. The proteins were boiled in SDS loading buffer before SDS-PAGE.

### Sirtuin activity assay

Epigenase Universal SIRT Activity/Inhibition Assay Kit (Epigentek, Cat # P-4036) was used as described in protocol. To detect the activation of SIRT7 by RSV, keratinocytes were pretreated with RSV or DMSO and then equal amounts of SIRT7 protein got by immunoprecipitation were added to reaction buffer in each well. To detect whether RSV directly activates SIRT7, equal amounts of purified SIRT7 protein from in vitro-expression were incubated with RSV or DMSO in reaction buffer in each well. The absorbance of the samples was detected at 450 nm with reference wavelength of 655 nm.

### In vitro deacetylation assay

HaCaT keratinocytes were treated with Nicotinamide (NAM) to induce HMGB1 acetylation, then HMGB1 was purified from cells by immunoprecipitation with protein A/G magnetic beads (Thermo Scientific). The beads were washed in high-salt buffer [50 mM Tris-HCl (pH 8.0), 1 M NaCl, 0.2 mM EDTA and 10% Glycerol] before incubated with purified SIRT7 in deacetylation buffer [10 mM Tris-HCl (pH 8.0), 4 mM MgCl_2_, 0.2 mM DTT and 10% Glycerol] in the presence or absence of 5 mM NAD^+^ at 30 °C for 1 h. Finally, the incubated samples were dissolved on SDS-PAGE and analyzed by western blot.

### High-performance liquid chromatography-mass spectrometry (HPLC-MS) analysis

HPLC-MS analysis was performed as previously described [24]. A 20-μg sample of immunoprecipitated protein mix was separated by SDS-PAGE and stained with Coomassie brilliant blue R250 and then processed with the Trypsin Profile IGD Kit (Sigma, PP0100). The resulting digest was processed with ZipTip C18 (Merck Millipore, ZTC18S096) then subjected to analysis by Thermo Fisher Scientific orbitrap fusion LC-MS/MS in positive ion, linear, delayed-extraction mode. Calibration was carried out using a standard peptide mixture. The mass spectra were subjected to proteomic databases for searching with Proteome Discoverer v2.1 software (Thermo Scientific) and the results were analyzed by Xcalibur 2.0.

### Xenograft mouse model

Female athymic nude mice (BALB/C-nu/nu, 3–4 weeks old) for xenograft experiment were purchased from Guangdong Medical Laboratory Animal Center. In total, 1.0 × 10^7^ A375 cells were subcutaneously implanted into the left and right flanks of 4-week-old nude mice. Then both left and right tumors were exposed to IR treatment every other day (other parts covered with lead plates to avoid IR exposure), with a total dose of 12 Gy. In the meantime, 20 μM RSV was applied to the tumor on one side and solvent applied on the other side before and after IR treatment. The tumor diameters were recorded every other day to generate a tumor growth curve.

### Statistical analysis

Statistical tests were done for independent-samples using *t*-test or one-way ANOVA tests (SPSS v20). All statistical tests incorporated two-tailed tests and homogeneity of variance tests and were thought to indicate significant difference if *p < 0.05, **p < 0.01, or ***p < 0.001. Sample sizes referred to the general application of the field and were not statistically predicted.

## Results

### Resveratrol counteracts ionizing radiation-induced DNA damage in keratinocytes

**Fig. 1:**
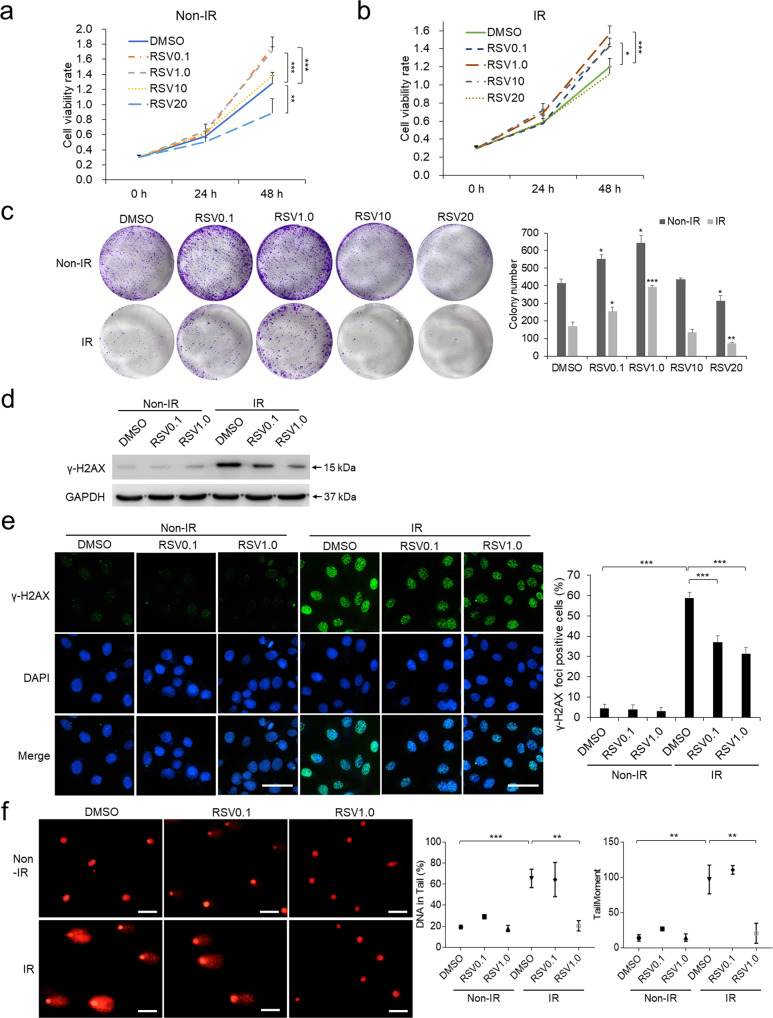
Resveratrol (RSV) counteracts ionizing radiation (IR)-induced DNA damage in keratinocytes. Measurements of cell viability by CCK-8 assay (**a**, **b**) and colony formation assay (**c**) were performed in keratinocytes pretreated with DMSO or different concentrations of RSV with control or IR treatments. **d** Detection of γ-H2AX by Western blot in keratinocytes pretreated with DMSO or different concentrations of RSV with control or IR treatments. **e** γ-H2AX staining was performed in keratinocytes pretreated with DMSO or different concentrations of RSV with control or IR treatments. γ-H2AX positive cells were count and statistically analyzed. Scale bar: 50 μm. **f** Comet assay in keratinocytes pretreated with DMSO or different concentrations of RSV with control or IR treatments. Scale bar: 100 μm. Each experiment was performed in triplicates and data are presented as mean ± s.d. *p < 0.05, **p < 0.01, ***p < 0.001.

To screen the pro-survival concentration of RSV in protecting skin from radiation injury, keratinocytes were pretreated with ascending concentrations of RSV from 0.1 to 20 μM and exposed to 8.0 Gy IR following the setting of experimental dose as previous reports [25, 26]. Cell viability assays showed that 0.1 and 1.0 μM RSV significantly enhanced cell viability while 10 μM RSV did not promote and 20 μM RSV even suppressed cell viability (Fig. 1a, b). We further analyzed cell survivability by performing colony formation assays after IR treatment. The result clearly demonstrated that keratinocytes pretreated with 0.1 and 1.0 μM RSV formed more colonies compared with control, indicating that 0.1 and 1.0 μM RSV can maintain cell survival in both Non-IR and IR treatment conditions (Fig. 1c).

Given that DNA strand break is one of the most serious biological effects induced by IR, we evaluated the effects of RSV against IR-induced DNA damage by detecting γ-H2AX. In total, 0.1 and 1.0 μM RSV pretreatments reduced the formation of nuclear γ-H2AX foci detected using immunofluorescence staining and γ-H2AX generation in Western blot, among which 1.0 μM RSV showed the strongest effects (Fig. 1d, e). Further, we performed comet assays and calculated the percentage of DNA in tail (the fraction of total DNA in the tail) and tail moment (the product of the tail length), which represented the degree of DNA breaks induced by IR treatment and were both decreased in keratinocytes pretreated with 1.0 μM RSV (Fig. 1f). Together, RSV pretreatment can rescue the decreased cell viability and suppress DNA damage induced by IR treatments in keratinocytes. Among all treatment groups, 1.0 μM RSV showed the strongest protection and thus we performed the following cellular studies using 1.0 μM RSV.

### The radioprotective effect of RSV is dependent on SIRT7

**Fig. 2:**
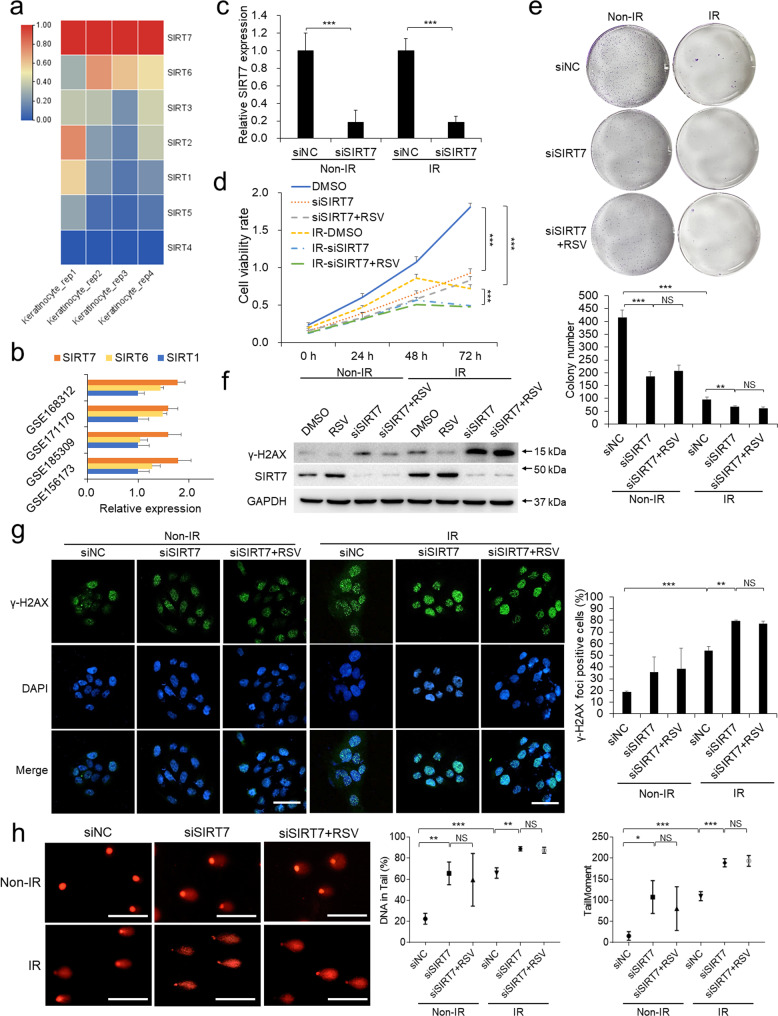
The radioprotective effect of RSV is dependent on SIRT7. **a** RNA sequencing was performed in HaCaT keratinocytes. **b** The relative expression levels of SIRT1, SIRT6 and SIRT7 from four GEO datasets based on transcriptomic sequencing of primary keratinocytes derived from human normal skin tissues. **c** SIRT7 was knocked down using siRNA and the relative SIRT7 expression was detected by qPCR. Measurements of cell viability by CCK-8 assay (**d**) and colony formation assay (**e**) were performed in keratinocytes pretreated with DMSO or 1.0 μM RSV and (or) siRNA targeting SIRT7 before IR treatment. **f** γ-H2AX and SIRT7 were detected by Western blot in keratinocytes pretreated with DMSO or 1.0 μM RSV and (or) siRNA targeting SIRT7 before IR treatment. **g** γ-H2AX staining was performed in keratinocytes pretreated with DMSO or 1.0 μM RSV and (or) siRNA targeting SIRT7 before IR treatment. Scale bar: 50 μm. **h** Comet assay in keratinocytes pretreated with DMSO or 1.0 μM RSV and (or) siRNA targeting SIRT7 before IR treatment. Scale bar: 100 μm. Each experiment was performed in triplicates and data are presented as mean ± s.d. *p < 0.05, **p < 0.01, ***p < 0.001.

As a STAC chemical, we supposed RSV may promote cutaneous radioresistance via sirtuins. To probe the major sirtuin member mediating the radioprotective role of RSV in keratinocytes, we first screened the expression levels of sirtuin members among four biological repeats of RNA-seq performed in HaCaT keratinocytes and observed SIRT7 is the highest-expressed sirtuin member among all seven human sirtuins (Fig. 2a). Moreover, analysis of four published GEO RNA-seq datasets (GSE168312, GSE171170, GSE185309, GSE156173) [27–30] of primary keratinocytes derived from human normal cutaneous tissues showed that SIRT7 is also the highest-expressed nuclear sirtuin among SIRT1, SIRT6 and SIRT7 (Fig. 2b). We hypothesized the highest-expressed SIRT7 should be most important for maintaining the cellular homeostasis, especially for genomic stability under IR circumstances in keratinocytes despite lack of depth exploration in previous reports.

To verify the radioprotective function of SIRT7 against CRI, we knocked down SIRT7 by RNA interference (Fig. 2c) and observed that loss of SIRT7 compromised cell viability under both non-IR and IR exposure conditions (Fig. 2d, e). Further, Trypan blue staining and Sytox Green (a nucleic dye penetrating dead cells while excluded by live cells) staining both showed that depletion of SIRT7 induced significant increase of cell death rate (Supplementary Fig. S1a, b). Moreover, SIRT7 knockdown resulted in higher γ-H2AX positive cell rates and generation of γ-H2AX in keratinocytes (Fig. 2f, g). Comet assays showed DNA portions in tail and tail moment were enhanced after the SIRT7 depletion (Fig. 2h). Consistently, SIRT7 overexpression (Supplementary Fig. S1c) alleviated the decrease of cell viability and DNA damage while partially compromised the cell death induced by IR-exposure (Supplementary Figs. S1d–g and S2a–c), supporting SIRT7 is critical for DNA damage repair and cell survival. At the same time, we also evaluated and observed the significant loss of the protective efficacy of RSV after SIRT7 depletion (Fig. 2d–h).

Collectively, SIRT7 is indispensable for the protective function of RSV in keratinocytes, indicating that the critical role of RSV depends on SIRT7.

### RSV enhances the deacetylase activity of SIRT7 via AMPK-mediated SIRT7 phosphorylation

**Fig. 3:**
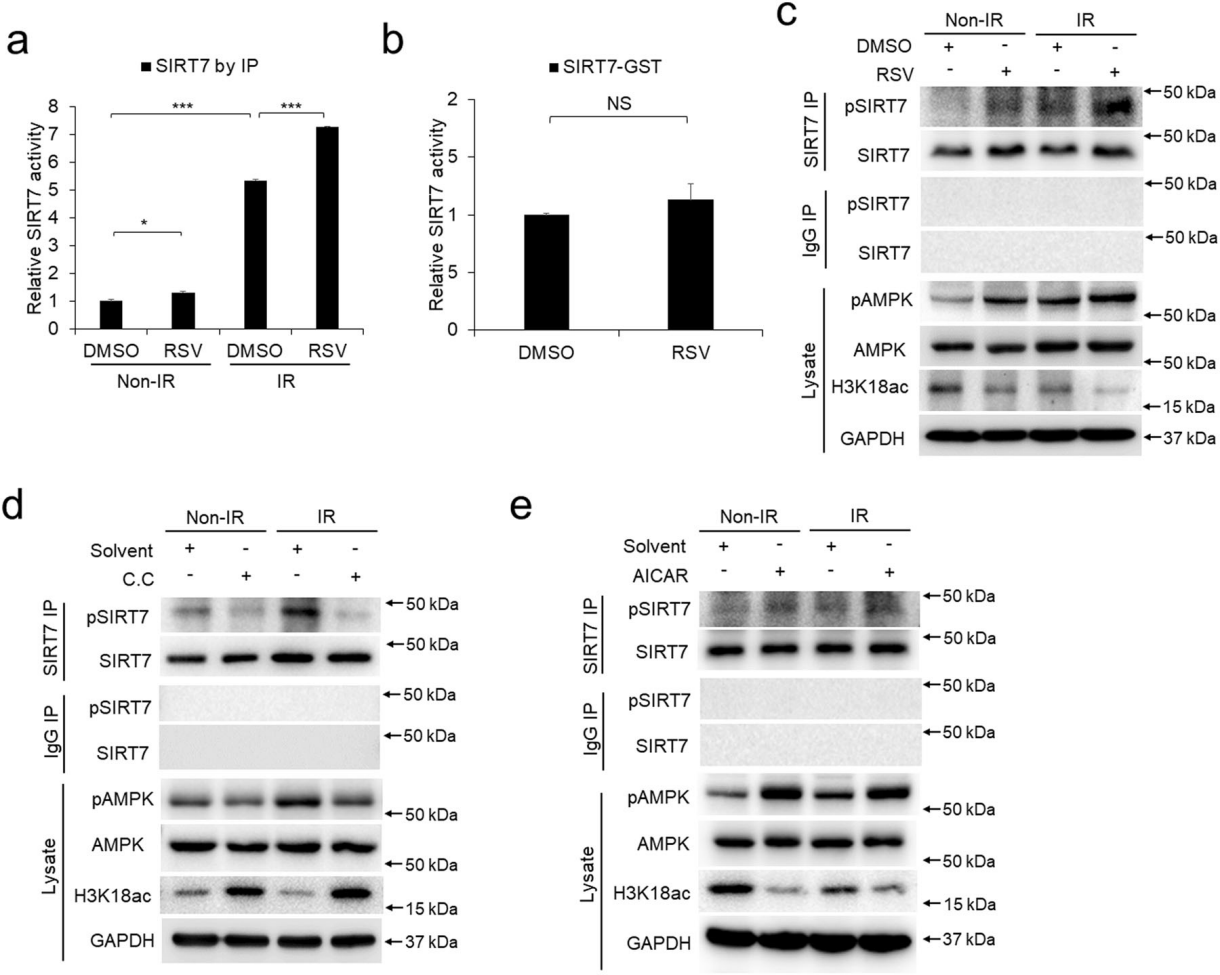
RSV enhances the deacetylase activity of SIRT7 via AMPK-mediated SIRT7 phosphorylation. **a** Detection of the deacetylase activities of immunopurified SIRT7 protein from control or IR-treated keratinocytes with or without the presence of RSV (*n* = 3). **b** Detection of the deacetylase activities of in vitro-expressed SIRT7 protein with or without the presence of RSV (*n* = 3). **c** Keratinocytes were treated with RSV before IR treatment and followed by immunoprecipitation using SIRT7 antibody and detected by Western blot. **d**, **e** Keratinocytes were treated with Compound C (10 μM, 6 h) and AICAR (1 mM, 12 h) before IR treatment and followed by immunoprecipitation using SIRT7 antibody and detected by Western blot. Each experiment was performed in triplicates and data are presented as mean ± s.d. *p < 0.05, **p < 0.01, ***p < 0.001.

To address whether RSV functionally regulates SIRT7, we checked in vitro activities of SIRT7 in the presence of RSV. IR treatment significantly increased the deacetylase activity of immunopurified cellular SIRT7 compared with control, which was further enhanced by RSV pretreatment (Fig. 3a). However, incubating RSV solely with purified SIRT7 protein from in vitro-expression did not significantly influence the deacetylase activities of SIRT7, indicating unknown factor(s) should function in mediating the activation of SIRT7 by RSV (Fig. 3b). As the activation of SIRT7 is tightly related with its phosphorylation [31], we further check the phosphorylation state of SIRT7. As predicted, IR and RSV both could promote the phosphorylation of SIRT7 (Fig. 3c).

Previous report has suggested AMPK is able to enhance RSV-regulated SIRT1 activation [32], we were curious to explore whether AMPK functions in mediating SIRT7 activation by RSV. Consistent with the above in vitro results, RSV enhanced the phosphorylation of SIRT7 and activated SIRT7 activity by showing the inhibited acetylation level of H3K18, a well-characterized substrate of SIRT7 (Fig. 3c). Meanwhile, the presence of RSV increased AMPK phosphorylation at Thr172 (pAMPK) without changing total AMPK protein level, which confirmed RSV functions in activating AMPK (Fig. 3c). Moreover, treatment with AMPK inhibitor Compound C (C.C) downregulated the phosphorylation of SIRT7 and increased the acetylation level of H3K18 (Fig. 3d), while AMPK activation by AICAR treatment upregulated the SIRT7 phosphorylation and decreased the acetylation level of H3K18 (Fig. 3e). In summary, the above results demonstrate that RSV enhances the phosphorylation and deacetylase activity of SIRT7 via AMPK activation.

### SIRT7 promotes DNA damage repair by interacting with HMGB1

**Fig. 4:**
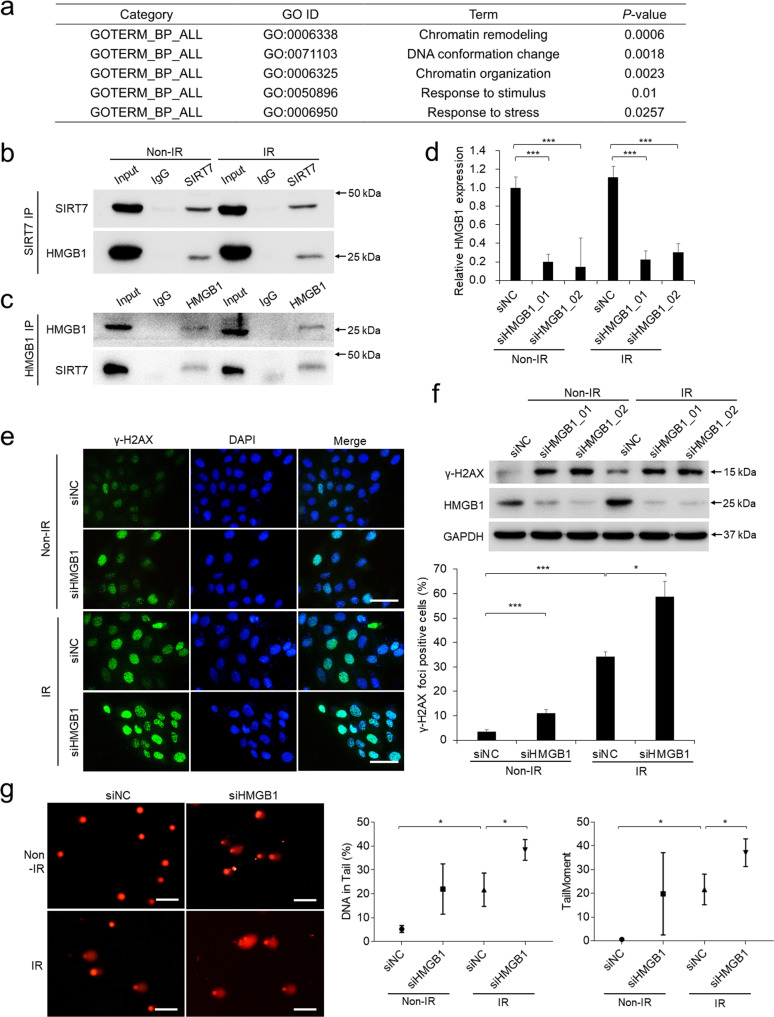
SIRT7 promotes DNA damage repair by interacting with HMGB1. **a** Gene Ontology (GO) analysis of the proteins specifically interacting with SIRT7 identified by HPLC-MS. **b**, **c** HMGB1 was verified to interact with SIRT7 by Co-IP followed with Western blot detection. **d** HMGB1 was knocked down using siRNA and the relative HMGB1 expression was detected by qPCR. **e** γ-H2AX staining was performed in keratinocytes pretreated with siRNA targeting HMGB1 before IR treatment. Scale bar: 50 μm. **f** Detection of γ-H2AX and HMGB1 was detected by western blot in keratinocytes pretreated with siRNAs targeting HMGB1 before IR treatment. **g** Comet assay in keratinocytes pretreated with siRNAs targeting HMGB1 before IR treatment. Scale bar: 100 μm. Each experiment was performed in triplicates and data are presented as mean ± s.d. *p < 0.05, **p < 0.01, ***p < 0.001.

To investigate how SIRT7 promotes DNA damage repair in keratinocytes, we performed immunoprecipitation of SIRT7 followed with HPLC-MS and tried to identify its interacting protein partners. Gene Ontology (GO) analysis of the specific binding protein profiles (Supplementary Table S3) generated the clustered GO categories including “Chromatin remodeling”, “DNA conformation change”, “Chromatin organization”, “Response to stimulus”, and “Response to stress” (Fig. 4a), which all contain one key protein HMGB1. There are total four peptides of HMGB1 detected in SIRT7-pulldown group compared with IgG-pulldown group from both control and IR treatments (Supplementary Fig. S3a, b). The physical interaction between SIRT7 and HMGB1 was confirmed by Co-IP assay (Fig. 4b, c). Nuclear HMGB1 has been suggested to act as DNA chaperone with DNA binding and bending activities and regulates key DNA-related events including DNA repair [33]. Since no report showing HMGB1 is involved in DNA damage repair in human keratinocytes, we validated it by RNA interference (Fig. 4d) and observed that loss of HMGB1 significantly exacerbated the DNA damage in keratinocytes (Fig. 4e–g). The above results strongly demonstrated that SIRT7 physically interacts with HMGB1 in keratinocytes and contributes to enhance DNA damage repair.

### SIRT7 deacetylates HMGB1 and redistributes HMGB1 to nucleus

**Fig. 5:**
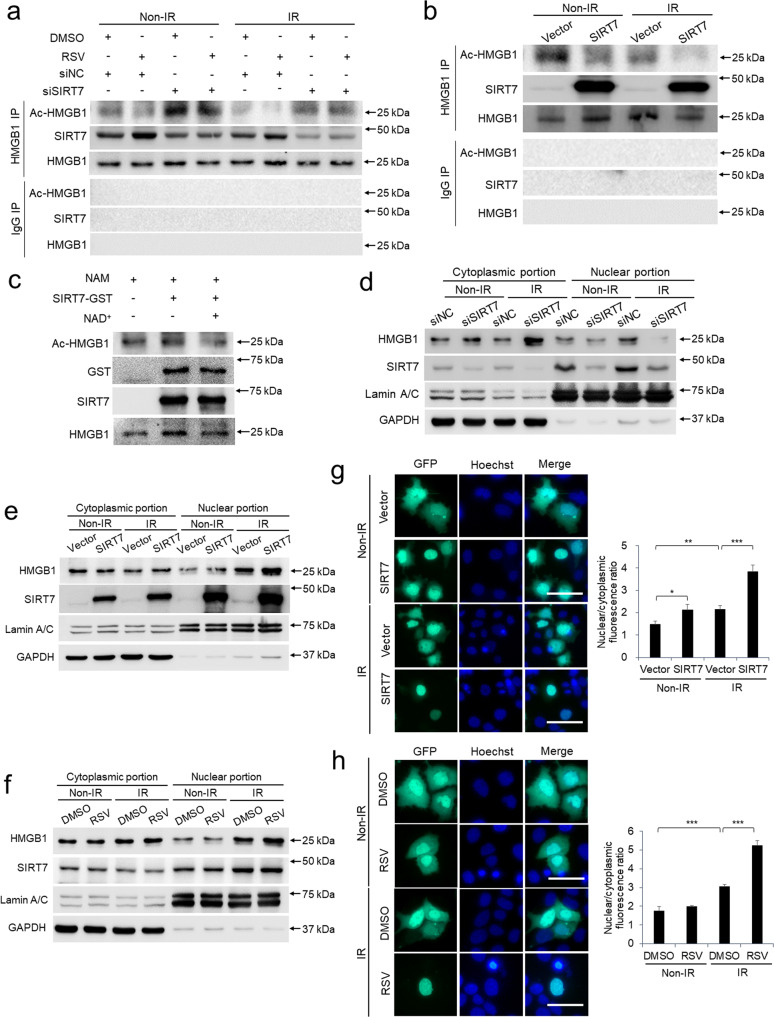
SIRT7 deacetylates HMGB1 and redistributes HMGB1 to nucleus. Immunoprecipitated HMGB1 acetylation was detected by Western blot in keratinocytes treated with 1.0 μM RSV and (or) siRNA targeting SIRT7 (**a**) or overexpression of SIRT7 (**b**) before IR treatment. **c** Immunopurified HMGB1 proteins from keratinocytes pretreated with NAM (10 mM, 6 h) were incubated with purified SIRT7 protein with or without NAD^+^ (5 mM). The acetylation levels were determined by immunoblotting using an anti–pan-lysine-acetylation antibody. HMGB1 and SIRT7 levels in cytoplasm and nucleus were detected by Western blot in keratinocytes after SIRT7 knockdown (**d**), SIRT7 overexpression (**e**) or RSV treatment (**f**). Detection of HMGB1-GFP cellular localization in keratinocytes after SIRT7 overexpression (**g**) or treated with RSV (**h**) before IR treatment. Scale bar: 50 μm. Each experiment was performed in triplicates and data are presented as mean ± s.d. *p < 0.05, **p < 0.01, ***p < 0.001.

The deacetylase nature of SIRT7 promoted us to probe whether SIRT7 regulates the deacetylation of HMGB1. Immunoprecipitation showed that IR-treatment reduced the acetylation degree of HMGB1 in keratinocytes, whereas knockdown of SIRT7 markedly increased the acetylation degree of HMGB1 (Fig. 5a). Moreover, RSV pretreatment promoted the association between SIRT7 and HMGB1 and reduced the acetylation degree of HMGB1 in siNC group but not in SIRT7 knockdown group (Fig. 5a), suggesting RSV-induced HMGB1 deacetylation is SIRT7-dependent. Consistently, SIRT7 overexpression reduced acetylation degree of HMGB1 in keratinocytes (Fig. 5b). To further verify the direct regulation of SIRT7 on HMGB1, in vitro deacetylation assay was performed following previous report [25]. SIRT7 alone did not change the acetylation degree of HMGB1, while the supplement of coenzyme NAD^+^ supported SIRT7 to significantly reduce the acetylation degree of HMGB1 (Fig. 5c). Taken together, RSV modulates the acetylation level of HMGB1 via SIRT7.

As the acetylation degree of HMGB1 determines its subcellular localization and corresponding functions [33], we wondered whether SIRT7-regulated HMGB1 deacetylation would influence the subcellular distribution of HMGB1. The total cellular proteins were fractionated into nuclear portion and cytoplasmic portion respectively for the following analysis. IR exposure led HMGB1 to be enriched in the nucleus while knockdown of SIRT7 induced a marked redistribution of HMGB1 to be mainly stay in the cytoplasm (Fig. 5d). Conversely, SIRT7 overexpression and RSV treatment could both promote cytoplasmic HMGB1 to transport into the nucleus (Fig. 5e, f). Interestingly, IR treatment also distributed SIRT7 into nucleus (Fig. 5f). We then utilized keratinocytes expressing HMGB1-GFP fusion protein and observed significantly enhanced nuclear redistribution of HMGB1 after SIRT7 overexpression and RSV treatment (Fig. 5g, h). In short, RSV promotes HMGB1 redistribution into nucleus to execute DNA damage repair via SIRT7.

### RSV rescues CRI in vivo by regulating AMPK/SIRT7/HMGB1 axis

**Fig. 6:**
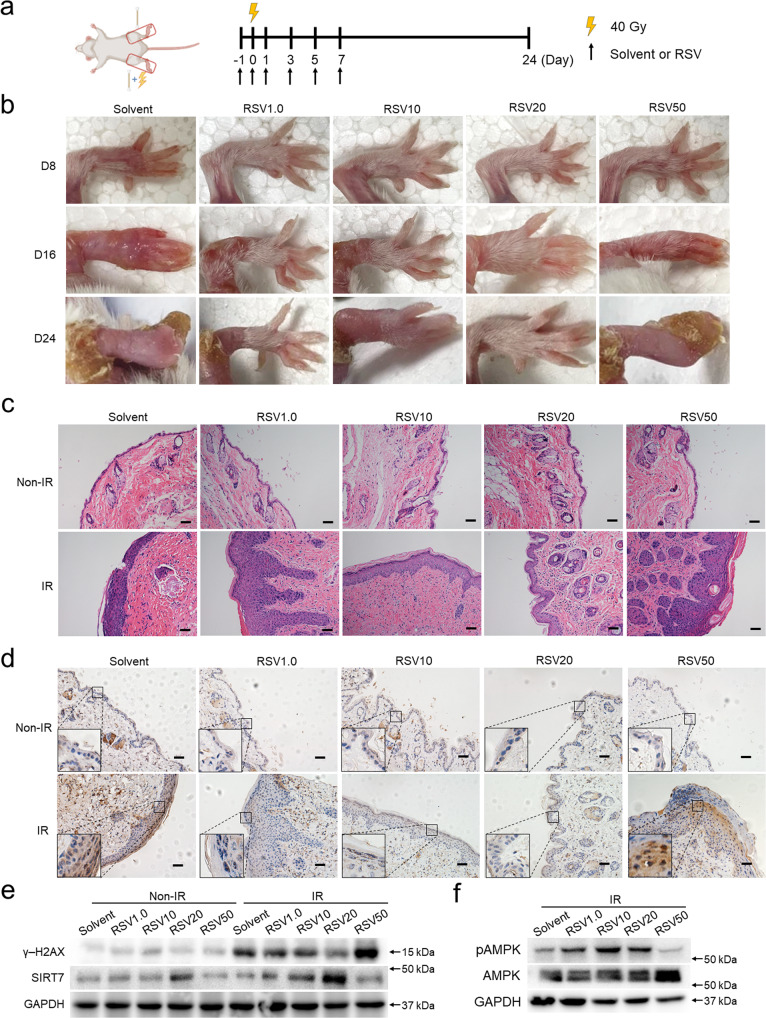
RSV alleviates radiation-induced skin injury in vivo. **a** Schematic illustration showing the time course to establish mice CRI model. **b** Representative CRI images of posterior limbs from 40 Gy X-ray-treated mice on day 8, 16, and 24. **c** Histological analysis of the cutaneous tissues from control and 40 Gy X-ray-treated mice. **d** IHC detection of γ-H2AX of the cutaneous tissues from control and 40 Gy X-ray-treated mice. Scale bar: 50 μm. γ-H2AX and SIRT7 (**e**) and pAMPK (**f**) were detected by Western blot in the cutaneous tissues from the cutaneous tissues from control and 40 Gy X-ray-treated mice.

**Fig. 7:**
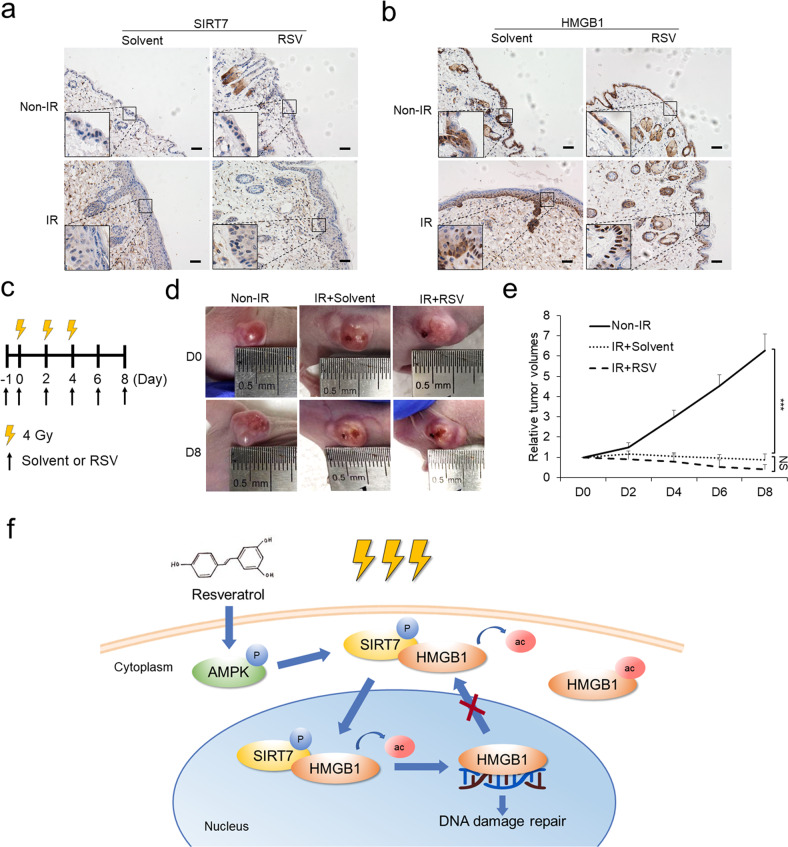
RSV rescues CRI in vivo by regulating AMPK/SIRT7/HMGB1 axis. IHC detection of SIRT7 (**a**) and HMGB1 (**b**) in the cutaneous tissues from mice posterior limb treated with solvent or 20 μM RSV and exposed to 40 Gy radiation. Scale bar: 50 μm. **c** Schematic illustration showing the time course for topical cutaneous application of RSV and IR exposure to subcutaneous xenografts. **d** Imaging of subcutaneous xenografts as control or treated with IR plus solvent or IR plus RSV on day 0 and day 8. **e** Tumor volumes (mm^3^) were recorded every 2 days. Tumor volume statistical data represent the average of independent experiments ± s.d., respectively. *p < 0.05, **p < 0.01, ***p < 0.001. **f** A model depicts the AMPK/SIRT7/HMGB1 regulatory axis that mediates the protective function of RSV to alleviate CRI.

To verify whether RSV-regulated AMPK/SIRT7/HMGB1 axis could be effective in vivo, a mice CRI model was established (Fig. 6a). We could observe the typical symptoms of CRI including aggravating erythema, desquamation, edema and ulceration gradually appeared and became gradually more serious with the time course in IR-treated solvent control group (Fig. 6b). At the same time, different dosages of RSV produced significant differential effects in rescuing CRI. In detail, 1.0 and 10 μM of RSV treatments could partially alleviate the damage while 20 μM RSV almost fully diminished the injury generated by IR exposure. Yet, it was hardly to observe effective rescue of the injury using 50 μM RSV (Fig. 6b), which suggests the curative effect of RSV is dose-specific. Epidermal thickening, angular cyst and glands degeneration were also observed in IR-treated solvent control group using H&E staining while 1.0 μM, 10 μM of RSV could alleviate these symptoms and 20 μM RSV treatment displayed the best-preserved skin structure (Fig. 6c). The results of γ-H2AX staining recapitulated the above results that 20 μM RSV treatment displayed the lowest staining intensity while 50 μM RSV treatment generated the strongest γ-H2AX intensity similar with solvent group (Fig. 6d), which is also supported by detecting the amount of γ-H2AX (Fig. 6e). Consistently, 20 μM RSV treatment showed the strongest effect in enhancing the generation of pAMPK and promoting SIRT7 expression (Figs. 6e, f and 7a). Further, 20 μM of RSV treatment promoted cytoplasm HMGB1 to redistribute into the nucleus as shown by immunochemistry staining (Fig. 7b), confirming that the role of RSV in reducing the degree of DNA damage largely attributed to SIRT7-regulated HMGB1 deacetylation and redistribution in vivo.

To exclude the possibility that topical application of RSV on skin potentially interfere the treatment effects of radiotherapy, we established a xenograft tumor model and simulated the process of radiotherapy (Fig. 7c). Growth curve analysis showed that radiotherapy significantly compromised xenograft tumor growth as predicted and treatment of 20 μM RSV displayed similar growth curve as solvent group (Fig. 7d, e). Such results exclude the possibility that cutaneous application of RSV would interfere the sensitization and efficacy of radiotherapy.

In summary, RSV can protect the skin from CRI by activating AMPK/SIRT7/HMGB1 axis and will not influence the curative effects of radiotherapy.

## Discussion

DNA damage is widely recognized as the one of the main biological effects induced by IR exposure [34], among which the direct damage generated by IR accounts for 30–40% and the indirect damage produced through free radicals accounts for 60–70% [35]. Cells respond to DNA damage by instigating a series of DNA damage repair signaling pathways, such as base excision repair, nucleotide excision repair, mismatch repair, homologous recombination and non-homologous end joining [36]. Failure of DNA damage repair will trigger the death of damaged cells, which ultimately lead to various injuries in tissues and organs.

Our in vivo and in vitro experiments indicated that RSV prevents DNA damage and alleviates CRI in a dose-specific manner. Similar dose-specific protective effects have been described in an ischemic heart model. RSV has been shown to be cardioprotective when rats were fed for 21 days with low doses (2.5 and 25 mg/kg), while the protective effect could not be observed in high doses (100 mg/kg) [37]. Low concentrations (1.0–10 mM) of RSV increased HO-1 expression via NF-κB and play a role in cardiovascular protection, whereas higher concentrations (≥20 mM) of RSV generated adverse effects [38]. In this study, we observed 1.0 μM RSV displayed the best radioprotective effect in vitro, while 20 μM RSV showed the best curative effects in protecting the cutaneous tissues from IR-induced injury in vivo. Taking into account the permeability of the stratum corneum, the resulted dose discrepancy between in vitro and in vivo studies may be primarily owing to the absorption rate of RSV at damage sites after topical cutaneous application.

Previous reports suggest RSV contributes to the maintenance of genomic stability through direct and indirect associations with target proteins [39]. For example, RSV inhibits oxidative DNA damage by reducing ROS levels in bronchial epithelial cells [40]. In 293T cells, RSV directly binds to the active site of TyrRS and stimulates the activation of PARP1, which promotes BRCA1-mediated homologous recombination repair [41]. However, the mechanism of RSV involved in DNA damage repair still lacks in-depth exploration, especially for skin. In this study, the highest-expressed nuclear sirtuin member in human keratinocytes, SIRT7, was identified to mediate the essential DNA damage repair function of RSV in skin. RSV enhances the deacetylase activity of SIRT7 under both control and IR exposure conditions. Moreover, when exposed to IR, the role of RSV to facilitate DNA damage repair and cell survival is dependent on SIRT7.

As a STAC, RSV is a recognized natural activator of sirtuin family members. Currently, RSV may activate sirtuins through two ways: direct activation by physical association with specific sirtuin or indirect activation of sirtuin through signaling pathways [42]. For example, RSV molecule is capable to directly bound to the N-terminal domain of SIRT1 and promote tighter binding between SIRT1 and its substrate to stimulate enzymatic activity [43]. RSV could also activate SIRT1 through increasing AMPK phosphorylation in C2C12 myotubes to promote muscle fiber type conversion from fast-twitch to slow-twitch [44]. Yet, there are still large gap before fully understanding how RSV activates other sirtuins and the following molecular events, especially for SIRT7. In this study, we did not observe significant direct activation of SIRT7 by RSV, suggesting that RSV activates SIRT7 mainly in an indirect manner. AMPK was next identified to be the key mediator of SIRT7 activation by RSV. In detail, RSV promotes AMPK phosphorylation to induce SIRT7 phosphorylation and enhance the deacetylase activity of SIRT7.

In the past decade, exploration the role and mechanism of SIRT7 in maintaining genome stability has become the focus of considerable interest [15]. For example, during early stages of DNA damage repair, SIRT7 influences the extent of histone H3K18 acetylation for efficient 53BP1 foci formation and facilitating overall NHEJ pathway-mediated DNA repair [26]. SIRT7 and DDX21 cooperate to prevent R-loop accumulation and thus safeguard genome integrity [45]. In late stage of DNA damage repair, SIRT7 deacetylates and transforms ATM into an inactive form, which is beneficial to timely mitigate DNA damage response [25]. Yet, how SIRT7 is involved in DNA damage repair in skin was not fully elucidated.

High-mobility group box 1 (HMGB1), the most abundant and well-characterized HMG protein, senses and coordinates the cellular stress responses and plays a critical role outside and inside of the cells [33]. The roles of HMGB1 are tightly related with its subcellular localization. Extracellular and cytoplasmic HMGB1 is involved in biological processes including inflammation, immunity and autophagy [33, 46]. Whereas, nuclear HMGB1 can directly associate to a variety of bulky DNA lesions and actively participate in DNA repair pathways including DNA mismatch repair, base excision repair, nucleotide excision repair and double strand break repair [47]. Interestingly, the subcellular localization of HMGB1 is determined by its acetylation status. For example, salidroside interrupts the release of HMGB1 out of nucleus via suppressing HMGB1 acetylation and thus inhibits inflammatory response [48]. In addition, autophagy-mediated HDAC inhibition promoted HMGB1 acetylation and resulted in HMGB1 being released to extracellular space in ferroptosis [49]. However, the relationship between the acetylation status of HMGB1 and DNA damage repair was rarely investigated. In this study, HMGB1 was identified to be a direct interacting partner of SIRT7, which was strengthened by RSV in skin. Under IR exposure condition, SIRT7 redirected HMGB1 into the nucleus via deacetylation and “switch on” its function for DNA damage repair. Our results provide the evidence that deacetylation of HMGB1 increases its DNA damage repair function and extend our understanding of the contribution of SIRT7 in maintaining genomic stability.

Collectively, our findings establish a novel AMPK/SIRT7/HMGB1 regulatory axis that functions in protecting skin from CRI. As depicted in Fig. 7f, RSV increases the deacetylase activity of SIRT7 via AMPK-mediated SIRT7 phosphorylation. The activation of SIRT7 directly deacetylates HMGB1 and redistributes HMGB1 into nucleus for participating DNA damage repair and protecting skin from CRI. Our findings extend the understanding of RSV in alleviating CRI and provides patients suffering from CRI during radiotherapy with a low-cost and high efficiency modality to improve the life quality.

## Supplementary information

Reproducibility checklist

Supplementary Data

Supplementary Table S1

Supplementary Table S2

Supplementary Table S3

Original Data

## Data Availability

RNA-seq data have been submitted in Gene Expression Omnibus (https://www.ncbi.nlm.nih.gov/geo/) with the accession code GSE202683. Mass spectrometry data have been deposited in ProteomeXchange (http://www.proteomexchange.org) with the accession code PXD033924. All data generated or analyzed during this study are included in this published article and its Supplementary Files are available from the corresponding authors on request.
