# Supplementary material for: Resveratrol rescues cutaneous radiation-induced DNA damage via a novel AMPK/SIRT7/HMGB1 regulatory axis
Source: Cell Death Dis. 2023 Jan 1;13(10):847. doi: 10.1038/s41419-022-05281-y (PMC9805450; doi:10.1038/s41419-022-05281-y)
Supplement: Supplementary file 2 — Supplementary Data [file 41419_2022_5281_MOESM2_ESM.pdf]

Supplementary Figure S1

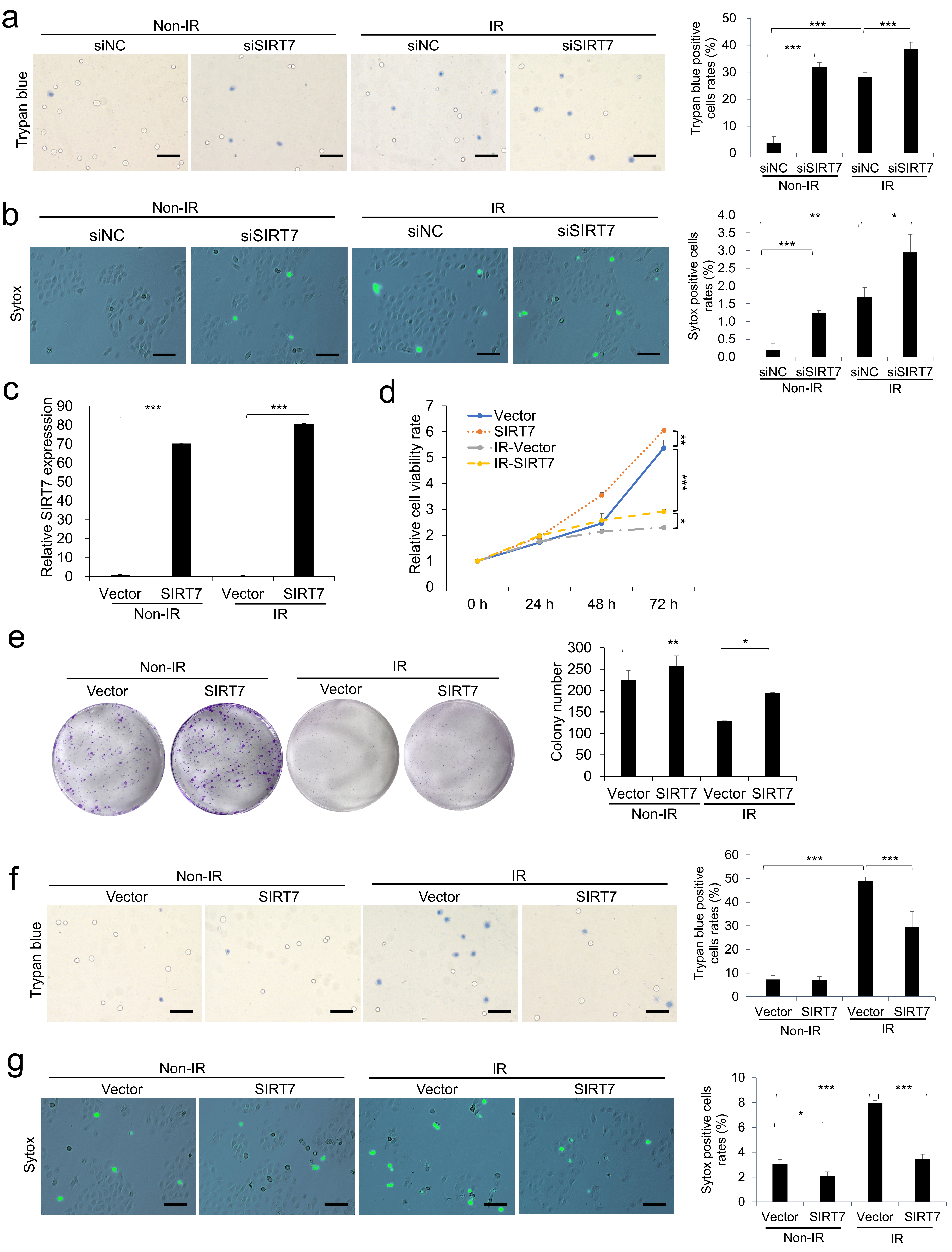

Supplementary Figure S1

Supplementary Figure S1. Trypan blue staining (a) and Sytox Green staining (b) was performed to detect the cell death rate in HaCaT keratinocytes treated with siSIRT7 before 8.0 Gy X-ray irradiation. Scale bar: 100 μm. (c) Overexpression of SIRT7 in keratinocytes and the relative SIRT7 expression was detected by RT-qPCR. Measurements of cell proliferation by CCK-8 assay (d) and colony formation assay (e) were performed in keratinocytes overexpressing SIRT7 after IR treatment. Trypan blue staining (f) and Sytox Green staining (g) was performed to detect the cell death rate in keratinocytes overexpressing SIRT7 after IR treatment. Scale bar: 100 μm. Each experiment was performed in triplicates and data are presented as mean ± s.d. *$P < 0.05$, **$P < 0.01$, ***$P < 0.001$.

Supplementary Figure S2

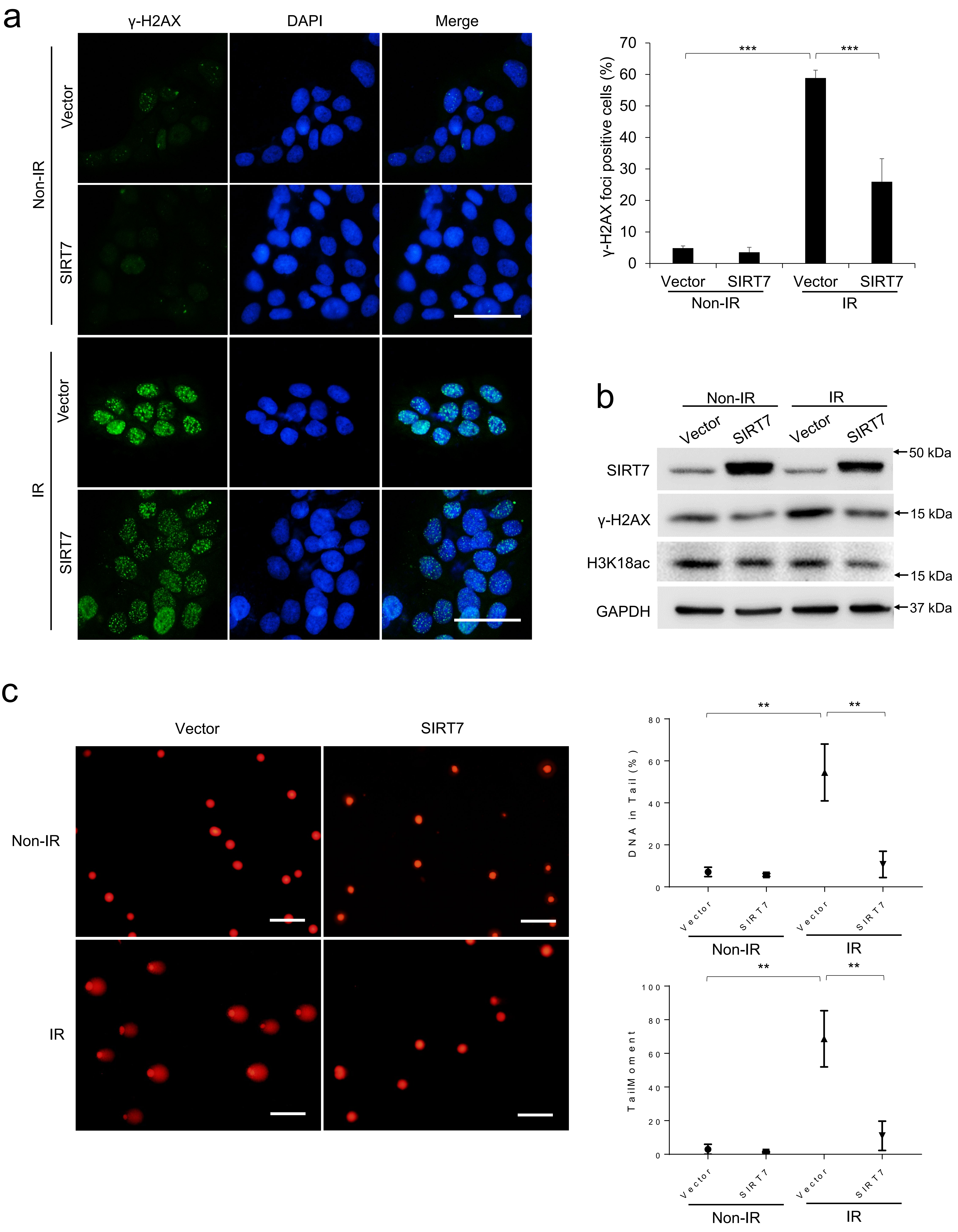

Supplementary Figure S2

Supplementary Figure S2. (a) γ-H2AX staining was performed in keratinocytes overexpressing SIRT7 after IR treatment. Scale bar: 50 μm. (b) γ-H2AX, SIRT7, H3K18ac was detected by Western blot in keratinocytes overexpressing SIRT7 after IR treatment. (c) Comet assay in keratinocytes overexpressing SIRT7 after IR treatment. Scale bar: 100 μm. Each experiment was performed in triplicates and data are presented as mean ± s.d. *$P < 0.05$, **$P < 0.01$, ***$P < 0.001$.

Supplementary Figure S3

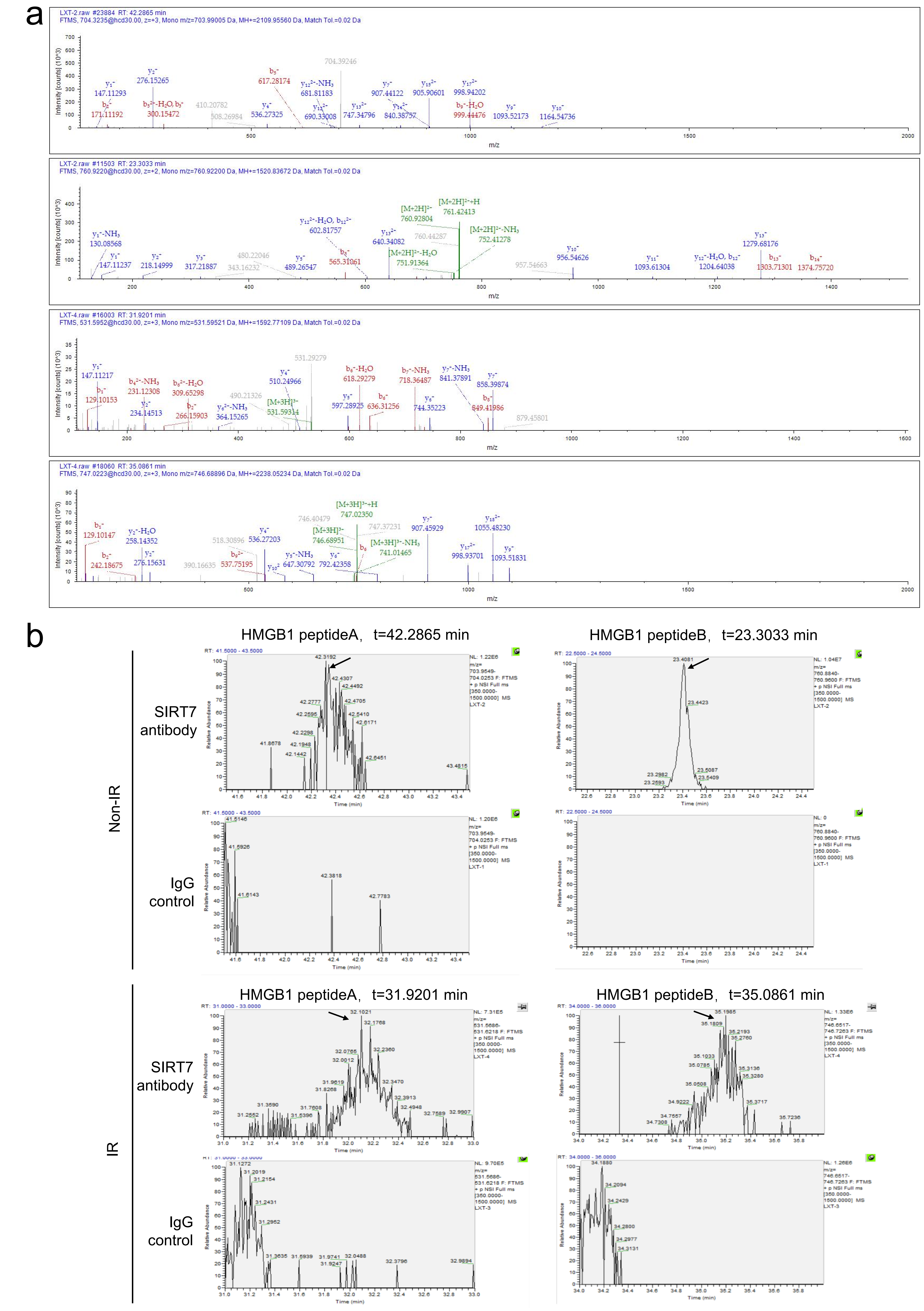

Supplementary Figure S3

Supplementary Figure S3. (a) Total four HMGB1 peptides were identified by HPLC-MS analysis of the protein mix immunoprecipitated by SIRT7 antibody under non-IR and IR treatment conditions respectively. (b) The chromatographic analysis of the protein mixes immunoprecipitated by SIRT7 antibody or control IgG. Arrows indicate the identified peaks of HMGB1 peptides in the samples pulled down by SIRT7, which are lack in corresponding control IgG samples.

Supplementary Figure S4

HaCaT cell line authentication

Ⅰ. Sample

Sample Name: labeled as 'HaCaT'

Ⅱ. Method and Procedure

1. PCR is amplified with STR Multi-amplification Kit (PowerPlex^{TM}16HS System);
2. PCR products are assayed with 3100 DNA Analyzer (Applied Biosystems®).
3. Amplification of gene COX1 and electrophoresis are employed to survey the species of the sample.

Ⅲ. Results

1. The STR profiles of the cell line sample are in the attached table and figure.
2. The search result in ATCC and DSMZ databases.
3. The electrophoresis figure of gene COX1.

HaCaT: ①Two loci have tri-alleles(D5S818 and CSF1P0). Contamination of other human cell line is not found (Figure 1 & Table 1). ②100% matched cell lines are not found in ATCC and DSMZ data banks (Figure 2 & Figure 3).③The sample is a human cell line. Contamination of other species cells are not found in the sample (Figure 4).

Operator: Xiaohua Mo　　Auditor: Xuanyi Liang

GUANGZHOU CELLCOOK BIOTECH CO.,LTD 15 OCT 2018 CELL STR PROFILING PASS

Guangzhou Cellcook Biotech Co., Ltd

Figure 1. STR profiles of HaCaT cell line

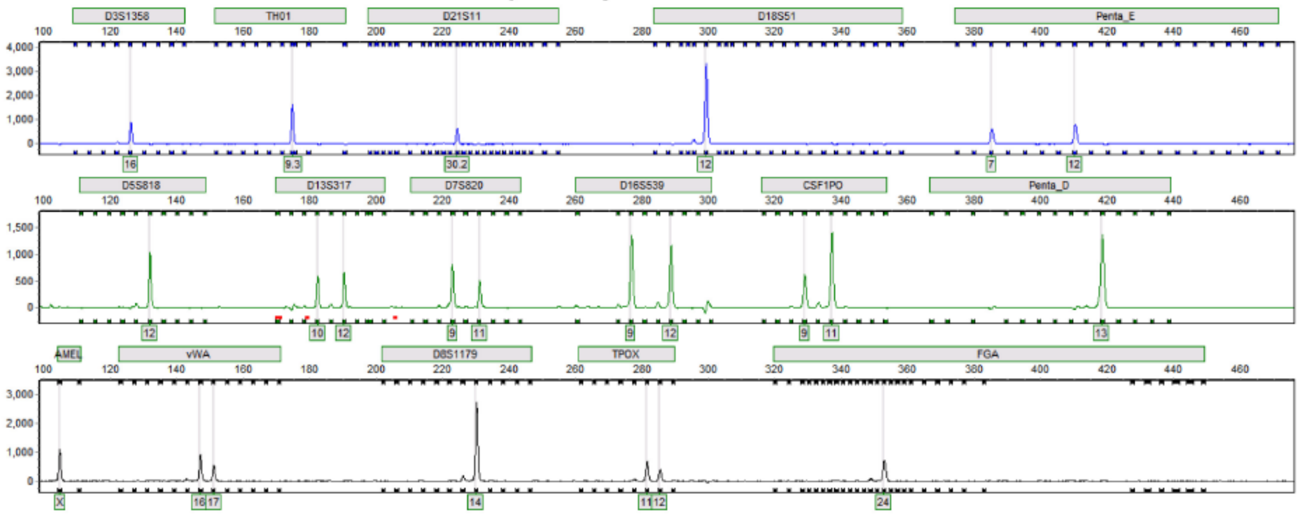

Table 1. STR profiles of HaCaT cell line

	Allele1	Allele2
D3S1358	16	
TH01	9.3	
D21S11	30.2	
D18S51	12	
Penta_E	7	12
D5S818	12	
D13S317	10	12
D7S820	9	11
D16S539	9	12
CSF1PO	9	11
Penta_D	13	
AMEL	x	
vWA	16	17
D8S1179	14	
TPOX	11	12
FGA	24	

Figure 2. Search result in ATCC database

SEARCH THE STR DATABASE

As part of our continuing efforts to characterize and authenticate the cell lines in the Cell Biology collection, ATCC has developed a comprehensive database of short tandem repeat (STR) DNA profiles for all of our human cell lines. View our brief tutorial before starting.

1. STR Profiling Analysis
2. Matching Algorithm
3. Interrogating the Database

Showing 1 - 1 Of 1　　PageSize: 100

Add to Cart	%Match	ATCC® Number	Designation	D5S818	D13S317	D7S820	D16S539	vWA	TH01	AMEL	TPOX	CSF1PO
☐	91.0	CRL-3298	MP46Uveal MelanomaHuman	12	10,12	11	12	16,17	9	X	11	11

Figure 3. Search result in DSMZ database

Result of STR matching analysis by your data.

- DSMZ Profile Database -

A graphical presentation is shown at the bottom of this page.

EV	Cell No.	Cell name	D5S818	D13S317	D7S820	D16S539	VWA	TH01	AM	TPOX	CSF1PO	Figures
	Query (Your Cell)		12	10,12	9,11	9,12	16,17	9.3	x	11,12	9,11	
1.09(36/33)	771	HACAT	12,12	10,12	9,11	9,12	16,17	9.3,9.3	X,X	11,12	9,11	-
0.85(28/33)	704	OAC-P4C	9,9	12,12	9,11	12,12	16,16	9,9	X,X	11,11	11,11	-
0.85(28/33)	CRL-1611	ACHN	12,12	12,12	9,11	12,13	16,17	8,8	X,X	8,11	11,11	-
0.85(28/33)	CRL-7826	Hs 38.T	11,11	12,12	9,9	9,9	17,17	9,9	X,X	11,11	11,11	-
0.85(28/33)	RCB1962	ACHN	12,12	12,12	9,11	12,13	16,17	8,8	X,X	8,11	11,11	-
0.79(26/33)	15	ML-2	12,12	9,12	9,11	9,12	16,16	7,9.3	X,X	8,10	10,11	-
0.79(26/33)	432	8-MG-BA	10,12	12,12	11,11	12,12	17,17	9,9.3	X,X	8,10	10,11	-
0.79(26/33)	661	UPCI-SCC-172	12,12	11,11	9,11	10,10	17,18	9.3,9.3	X,X	11,11	11,11	-
0.79(26/33)	CRL-10442	HCN-1A	11,12	11,12	11,12	12,12	17,17	9.3,9.3	X,X	11,11	10,10	-
0.79(26/33)	CRL-1593.2	U-937	12,12	10,12	9,11	12,12	15,15	9.3,9.3	X,X	8,11	12,12	-
0.79(26/33)	CRL-1855	LCL 721.221	12,12	11,12	10,11	12,12	14,17	9.3,9.3	X,X	8,12	11,12	-
0.79(26/33)	CRL-2367	TUR	12,12	10,12	9,11	12,12	14,16	6,9.3	X,X	8,11	12,12	-

Supplementary Figure S4

Supplementary Figure S4. The STR profiles of HaCaT cell line.
