# Supplementary material for: Resveratrol rescues cutaneous radiation-induced DNA damage via a novel AMPK/SIRT7/HMGB1 regulatory axis
Source: Cell Death Dis. 2023 Jan 1;13(10):847. doi: 10.1038/s41419-022-05281-y (PMC9805450; doi:10.1038/s41419-022-05281-y)

Original data

Related to Figure 1d

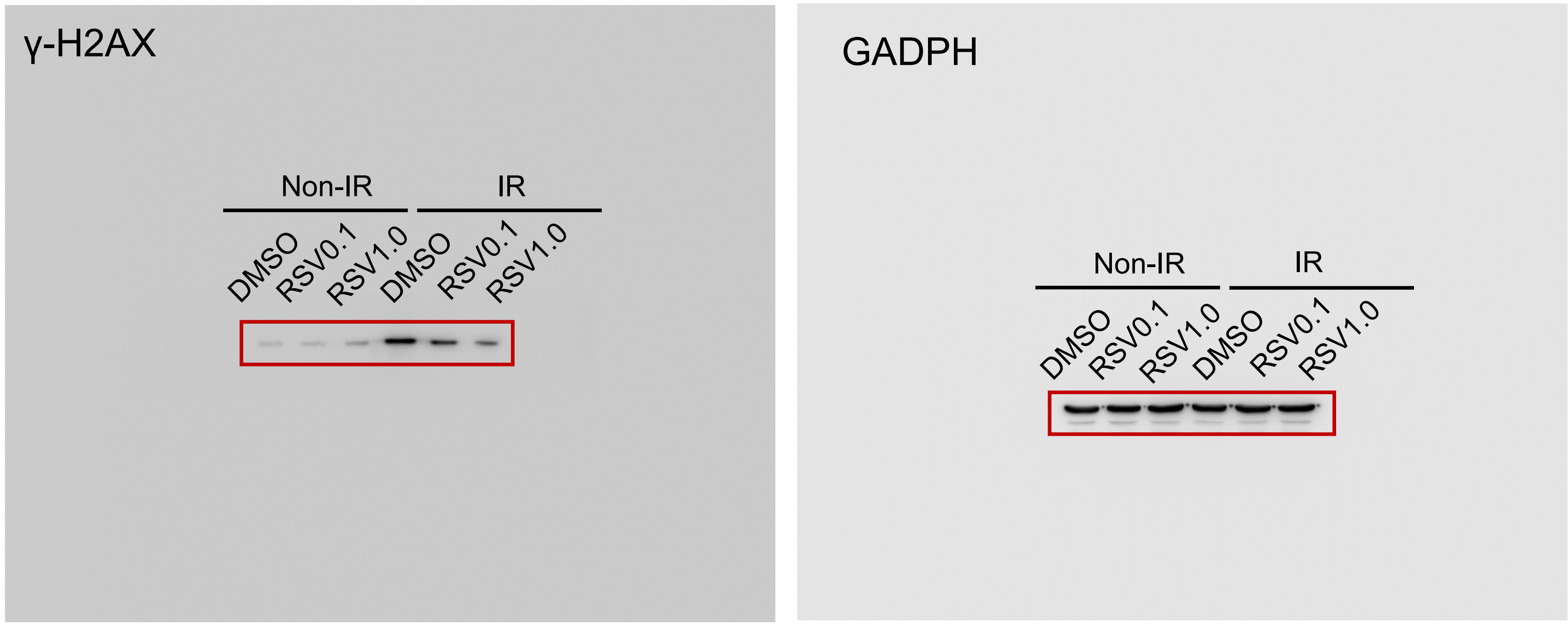

Related to Figure 2f

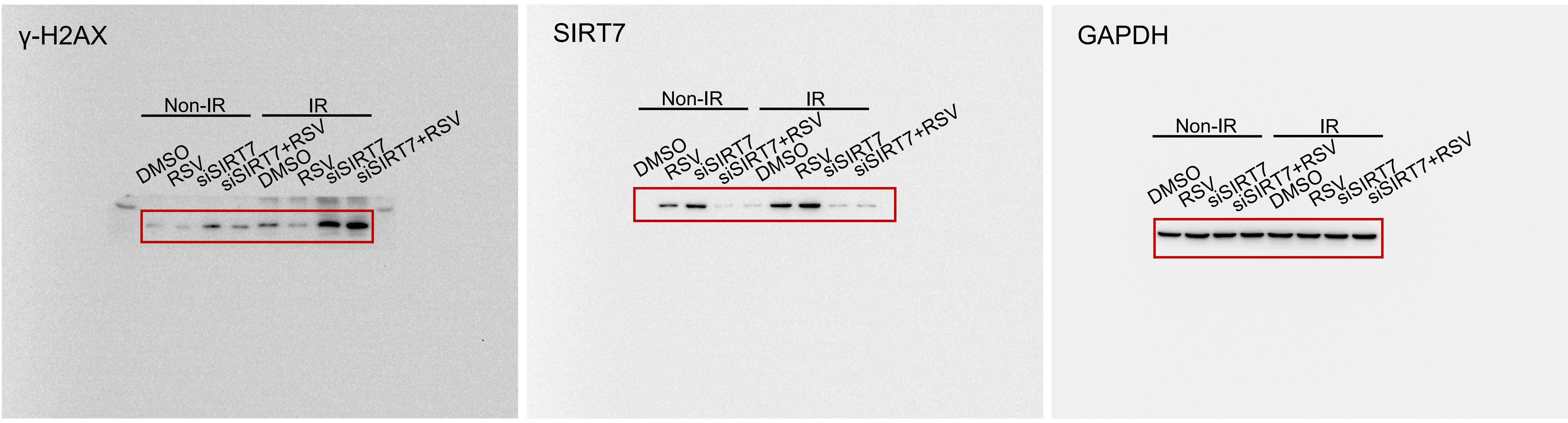

Related to Figure 3c

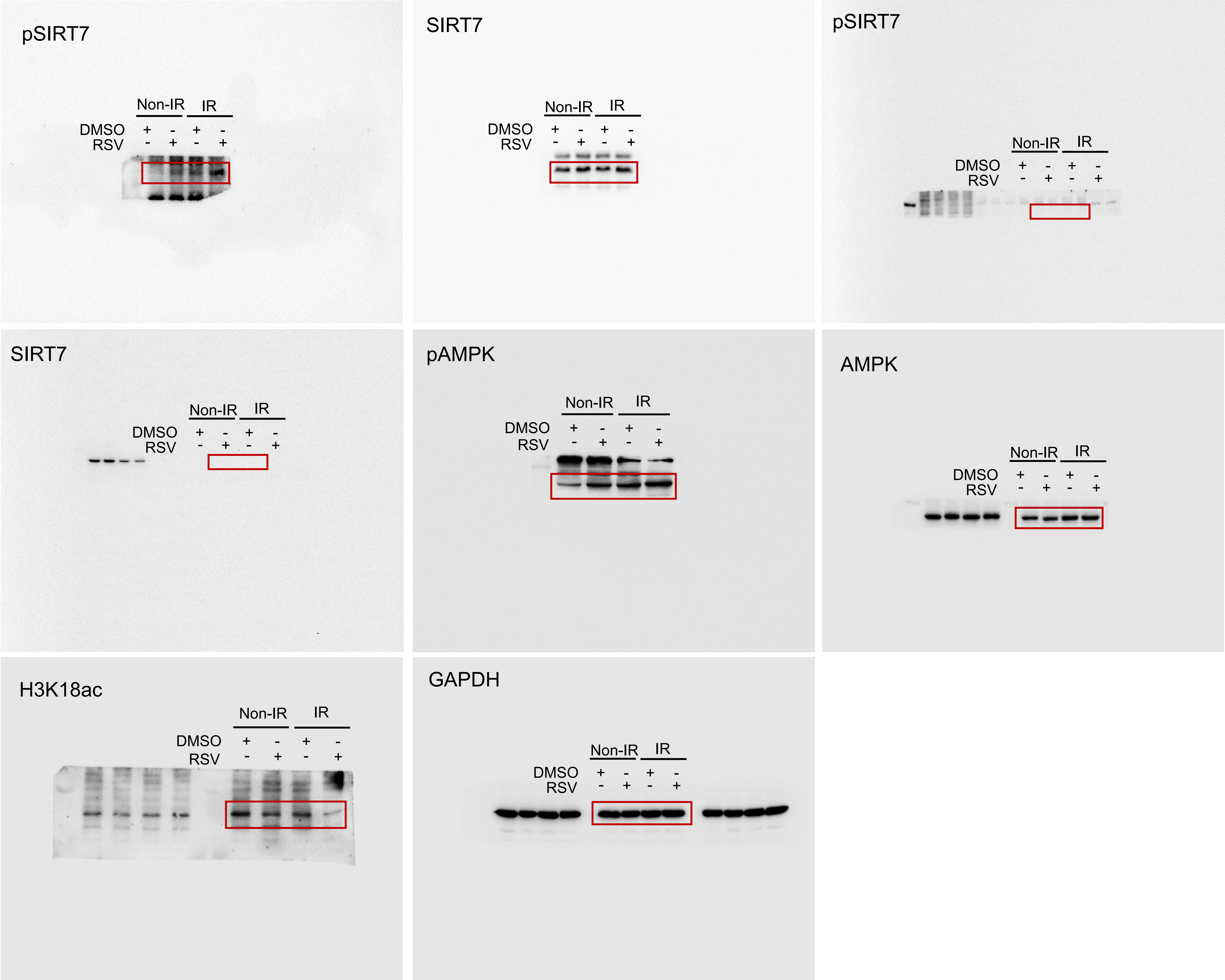

Original data

Related to Figure 3d

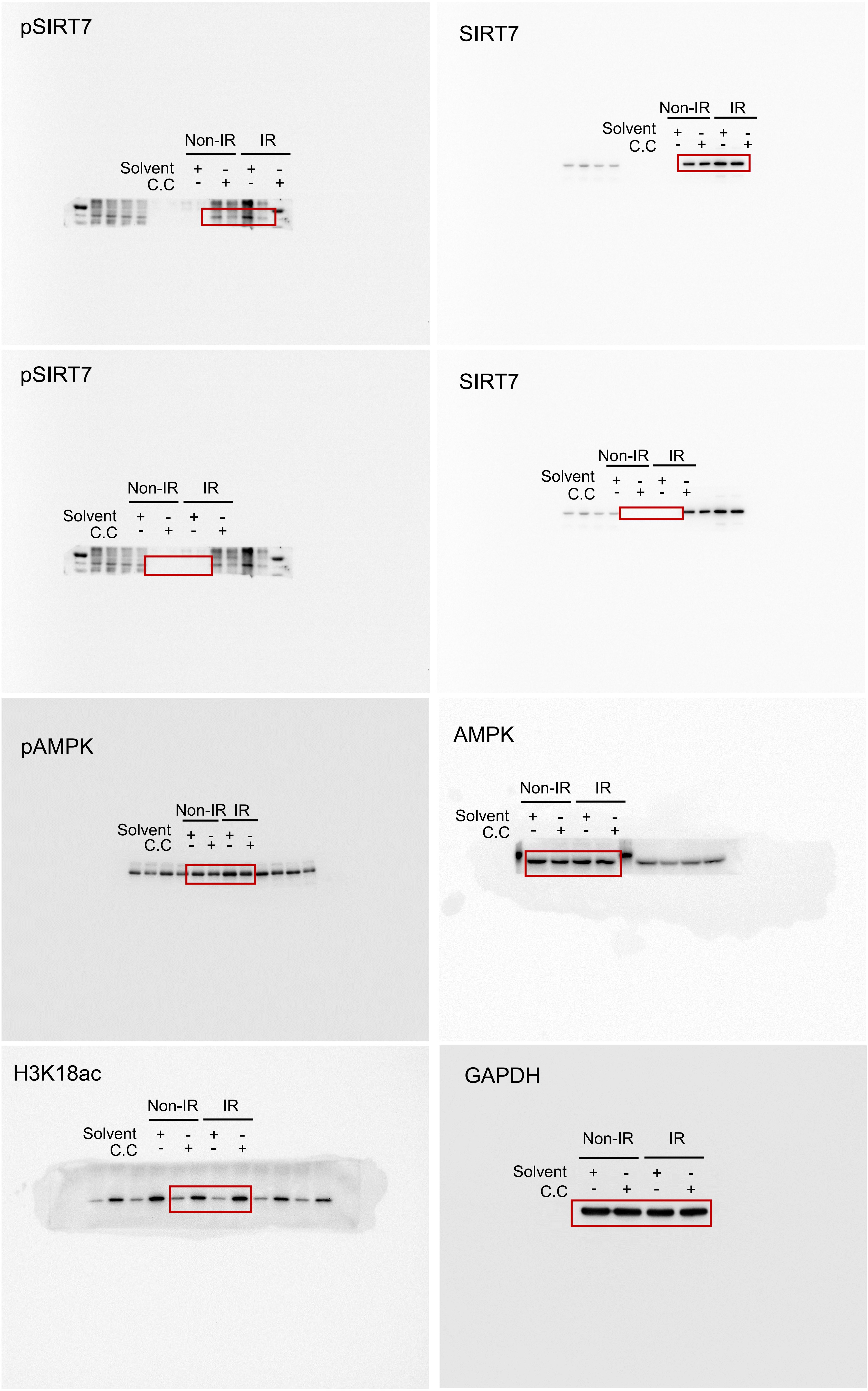

Original data

Related to Figure 3e

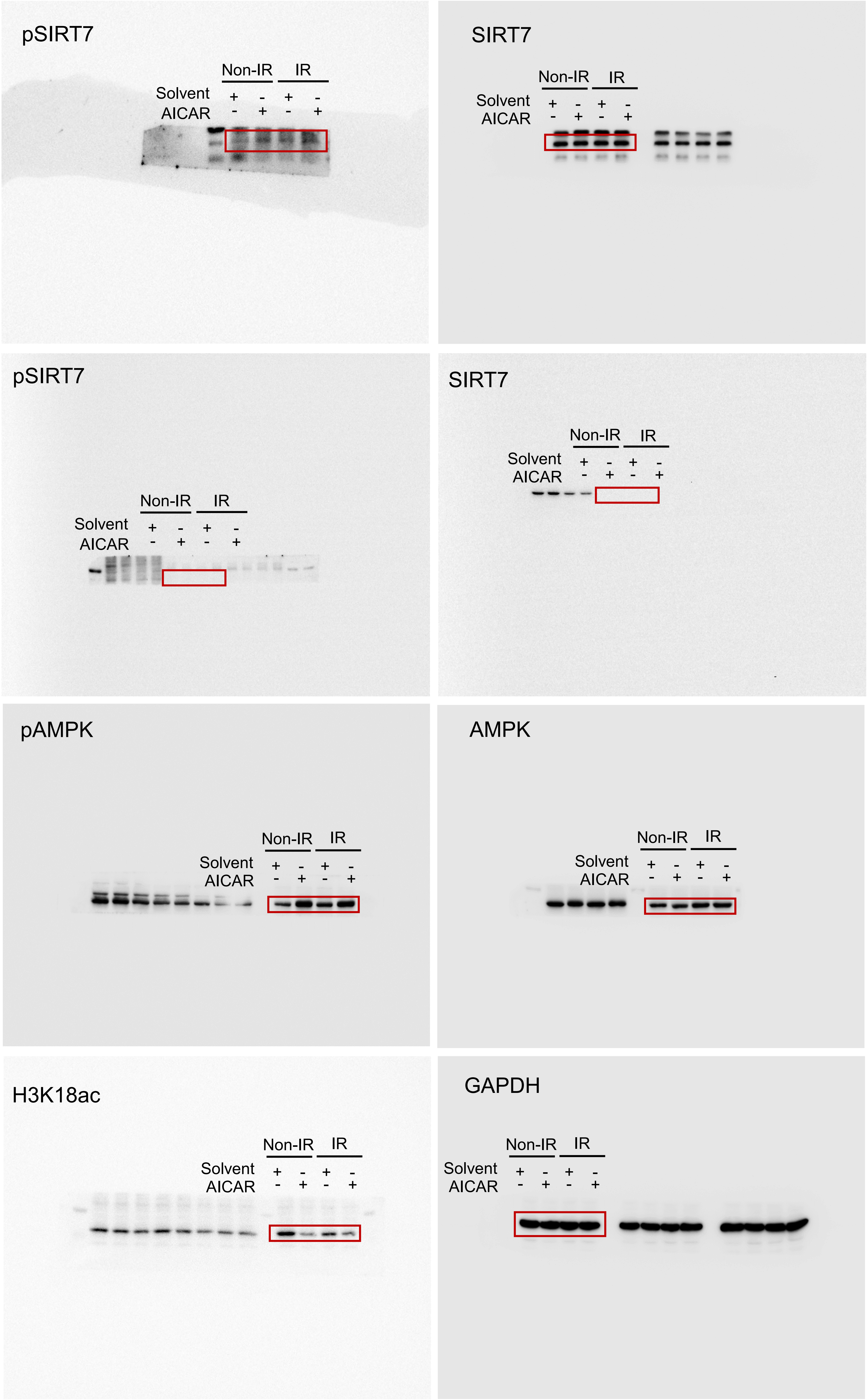

Original data

Related to Figure 4b

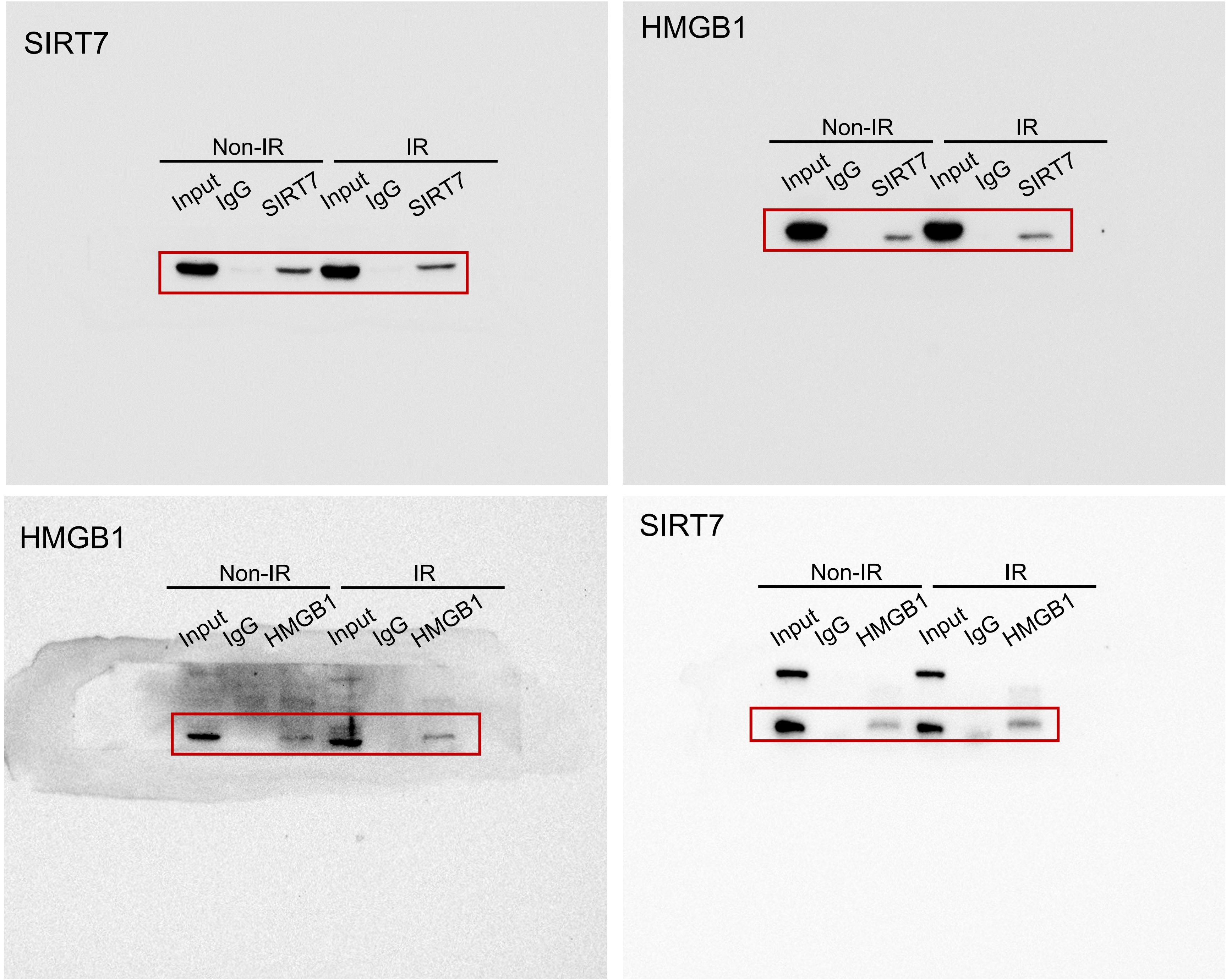

Related to Figure 4f

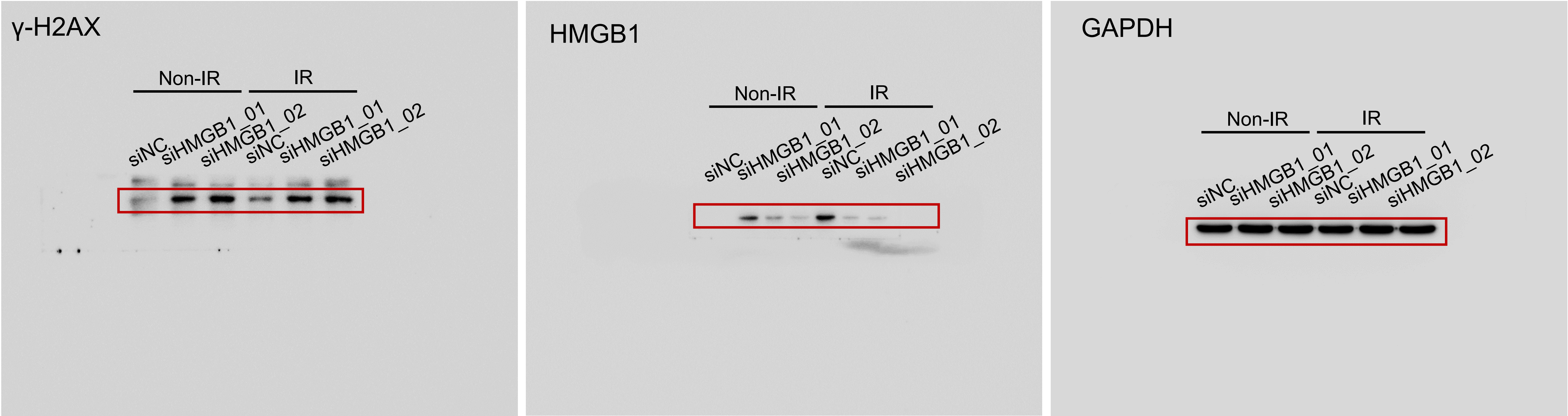

Related to Figure 5a

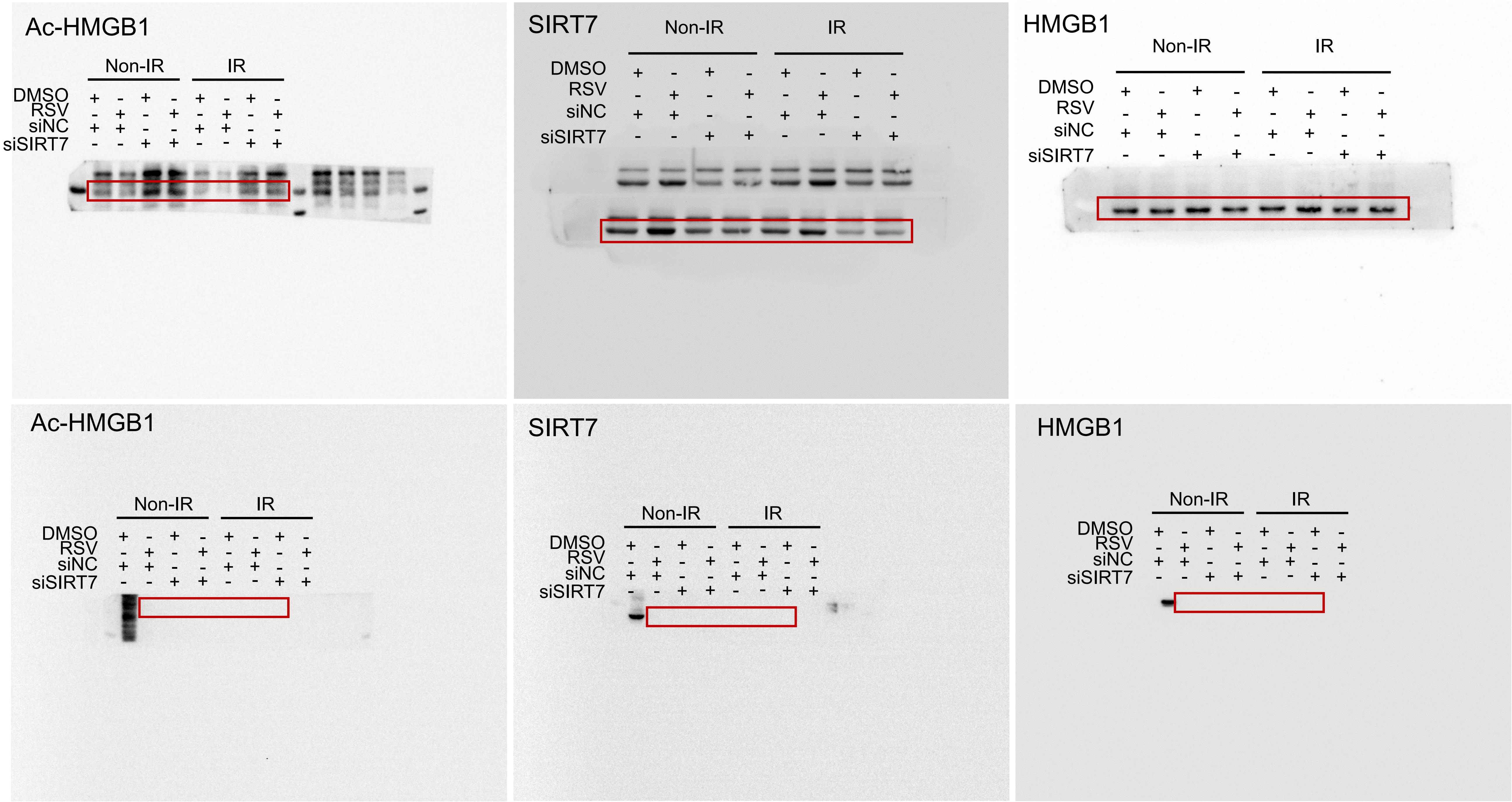

Original data

Related to Figure 5b

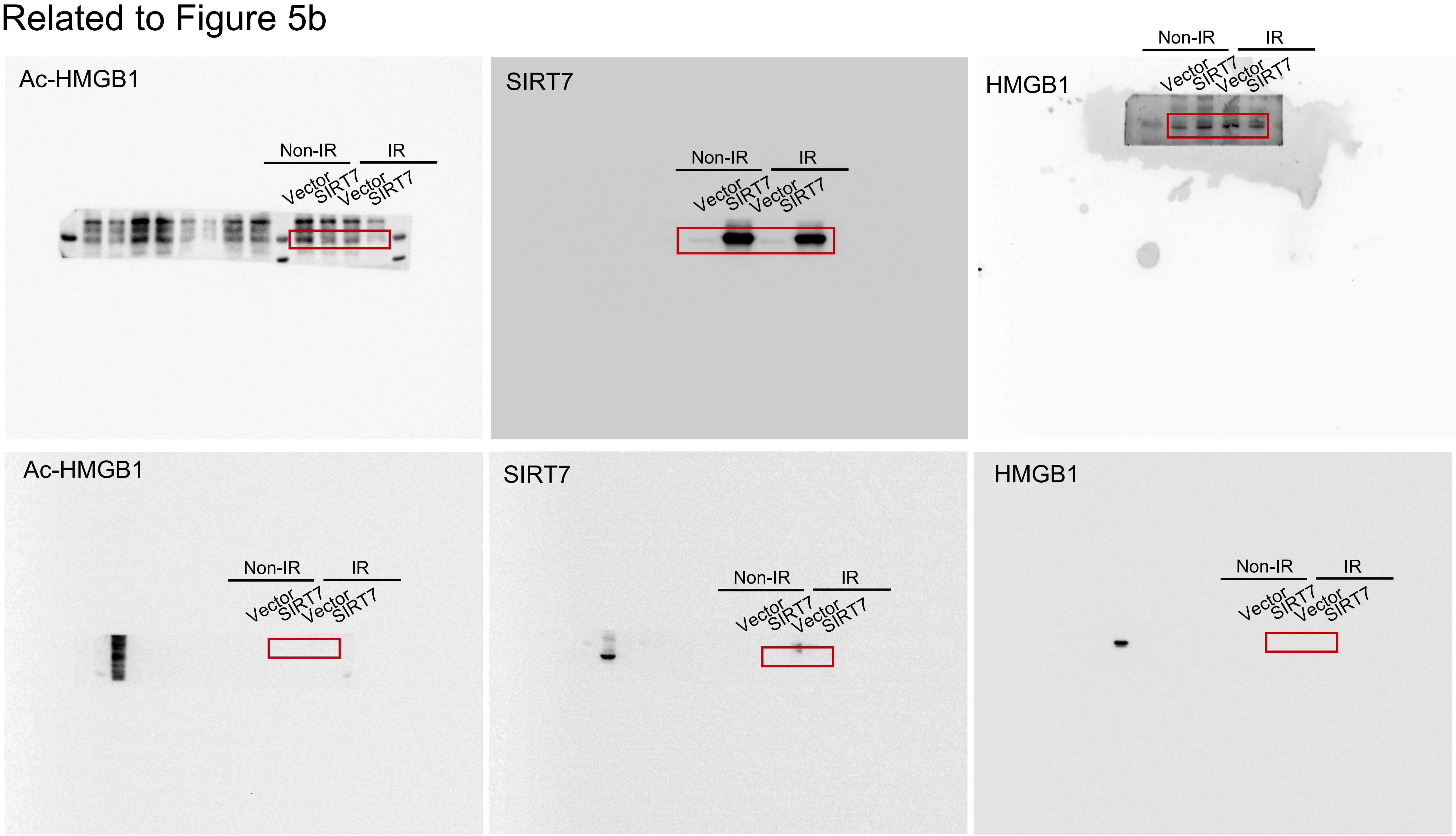

Related to Figure 5c

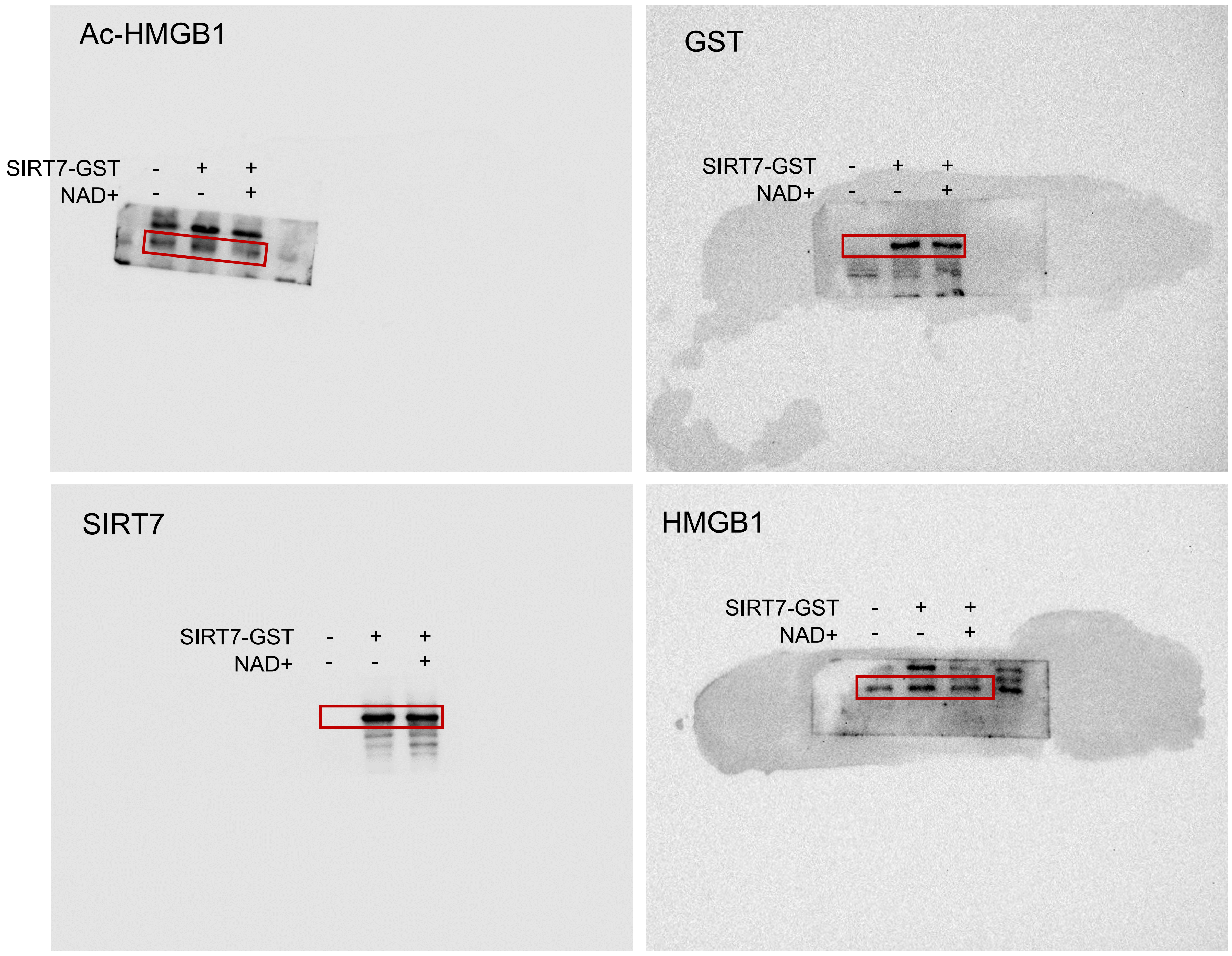

Original data

Related to Figure 5d

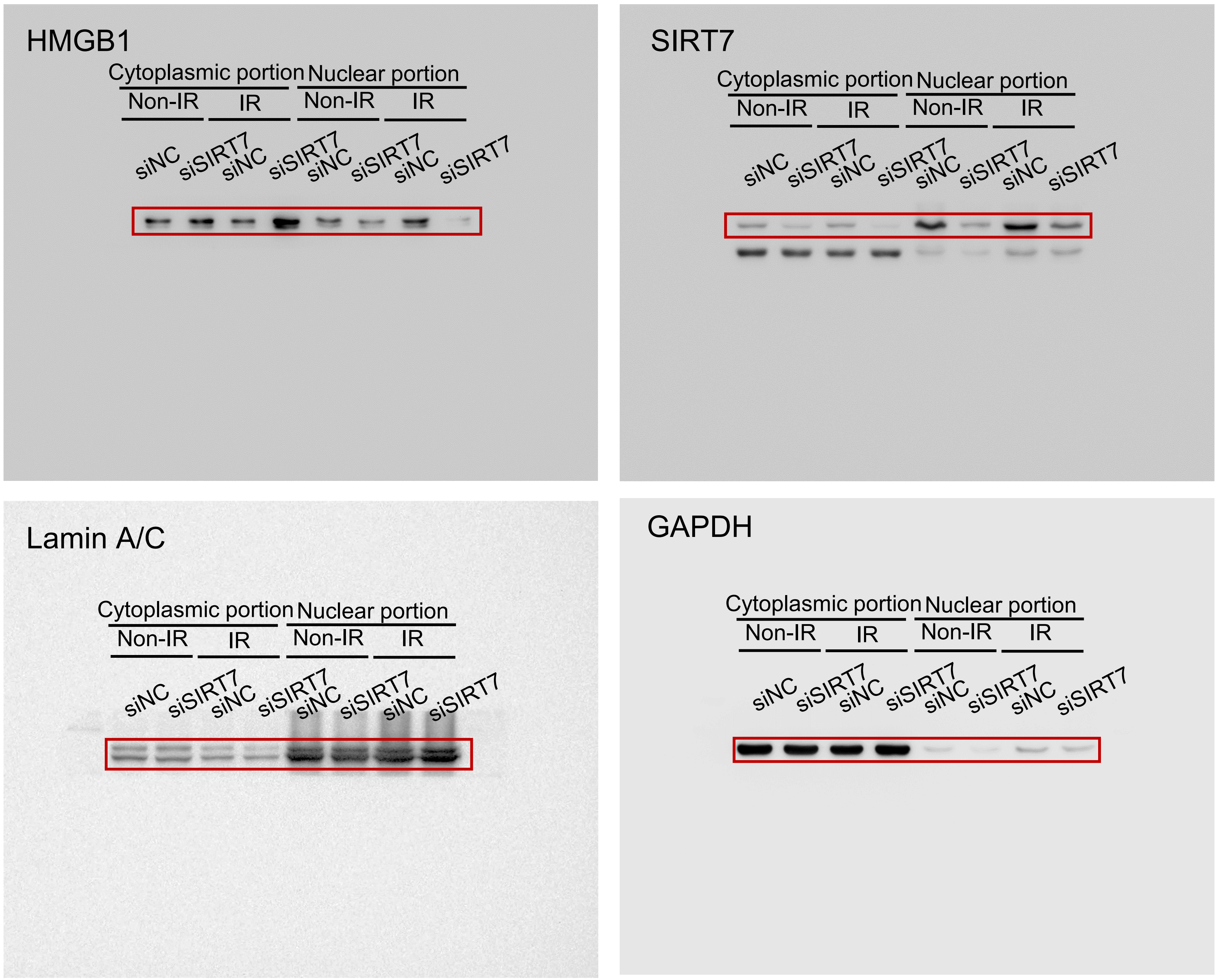

Related to Figure 5e

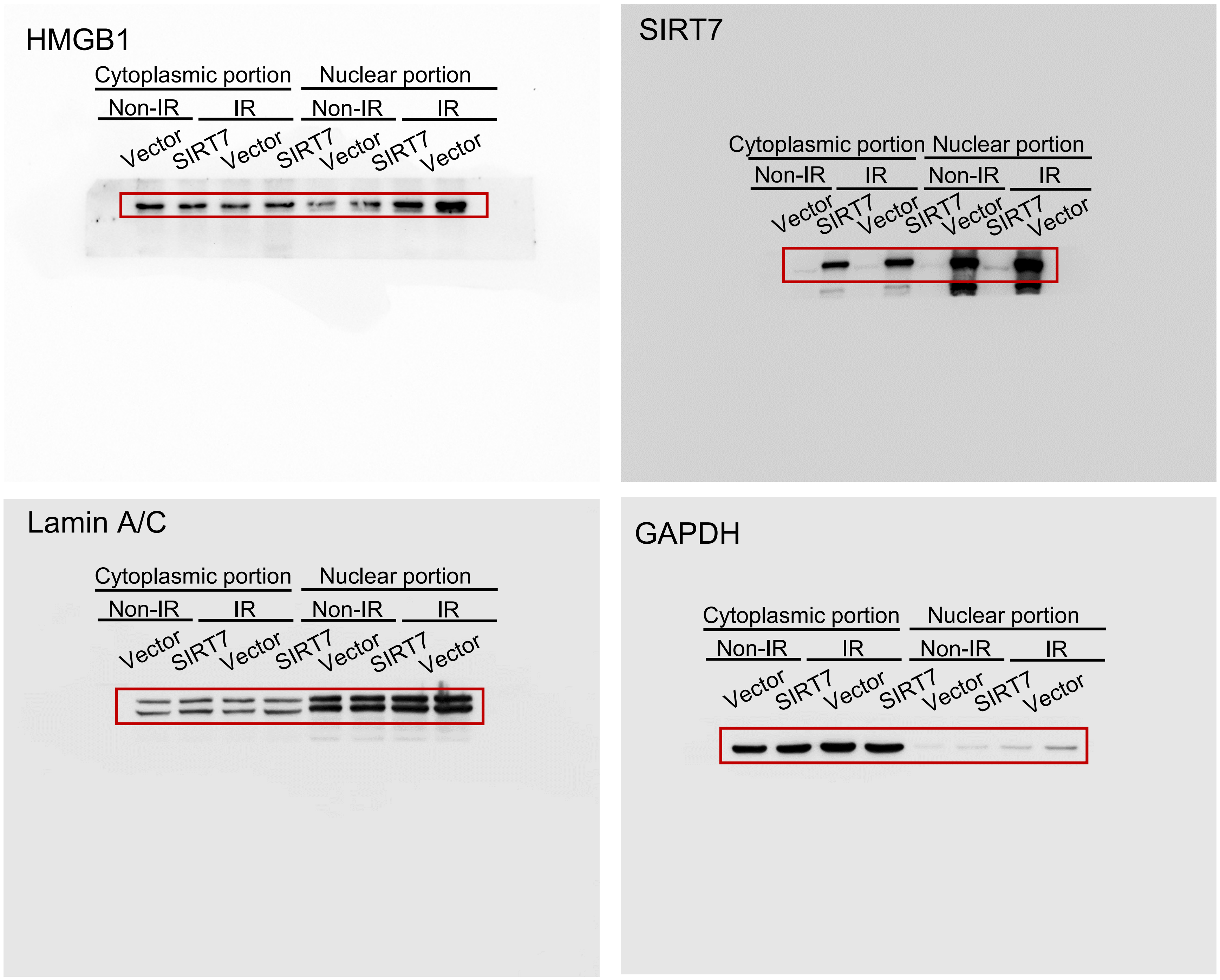

Original data

Related to Figure 5f

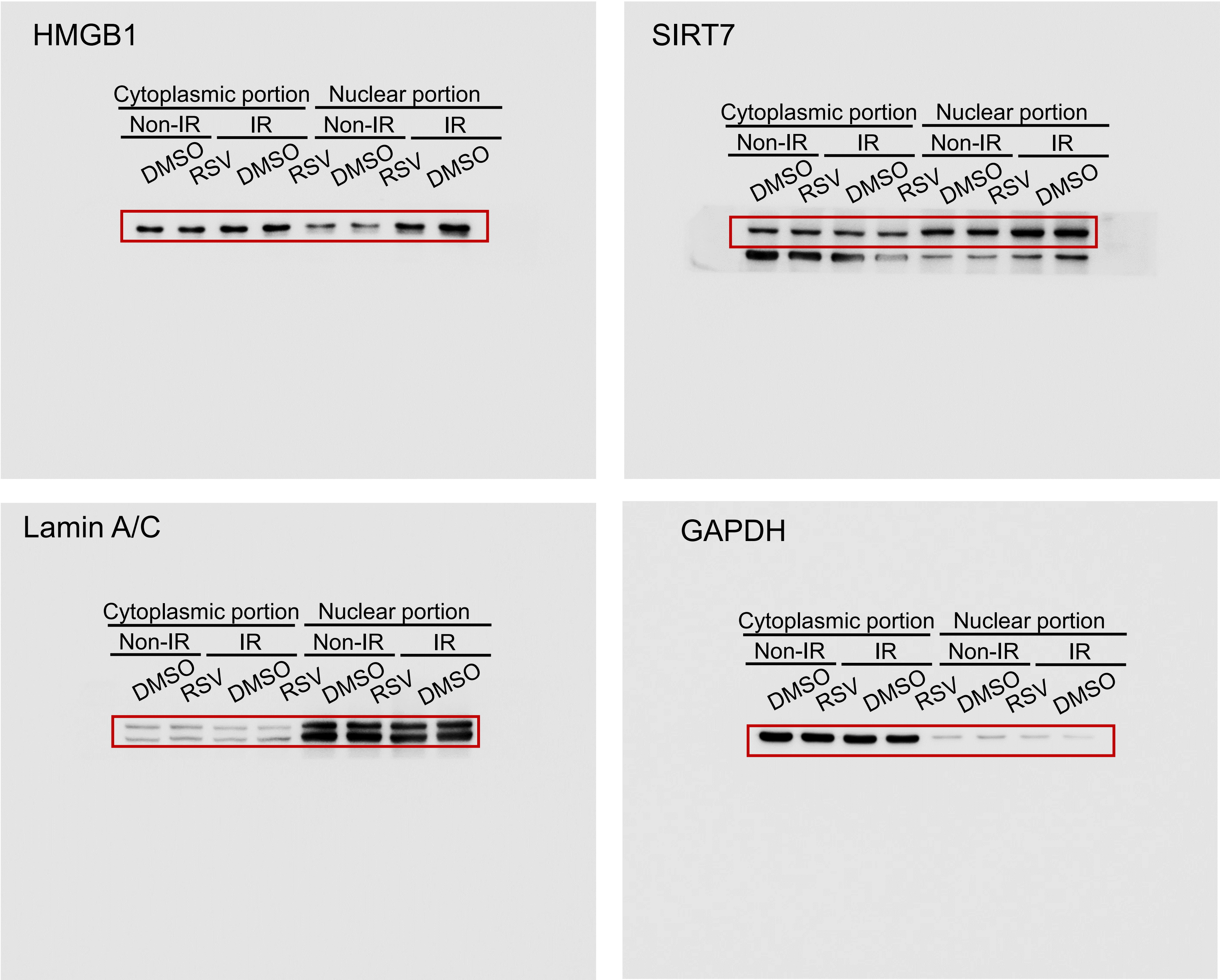

Related to Figure 6e

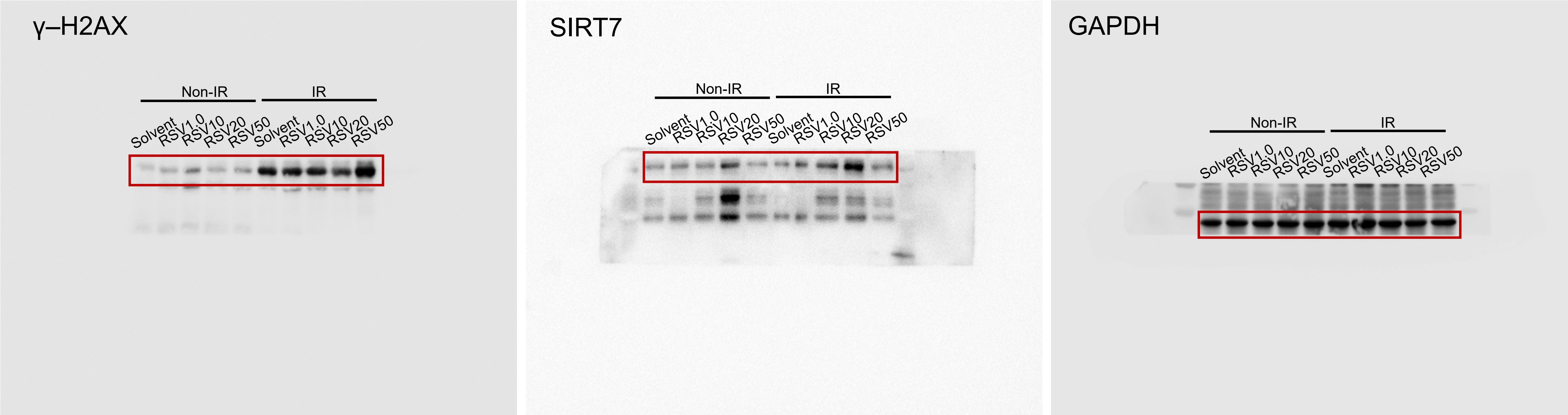

Original data

Related to Figure 6f

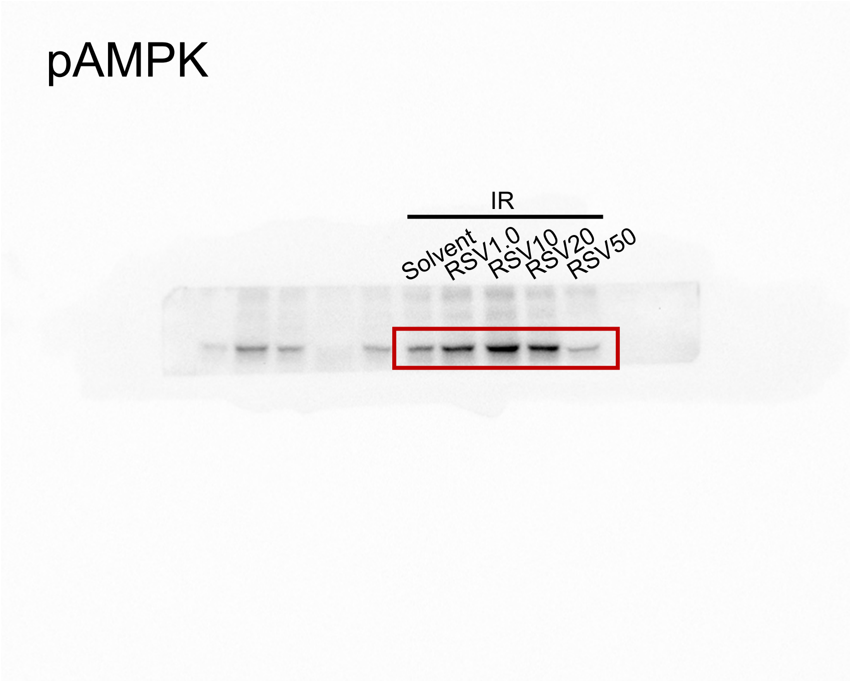

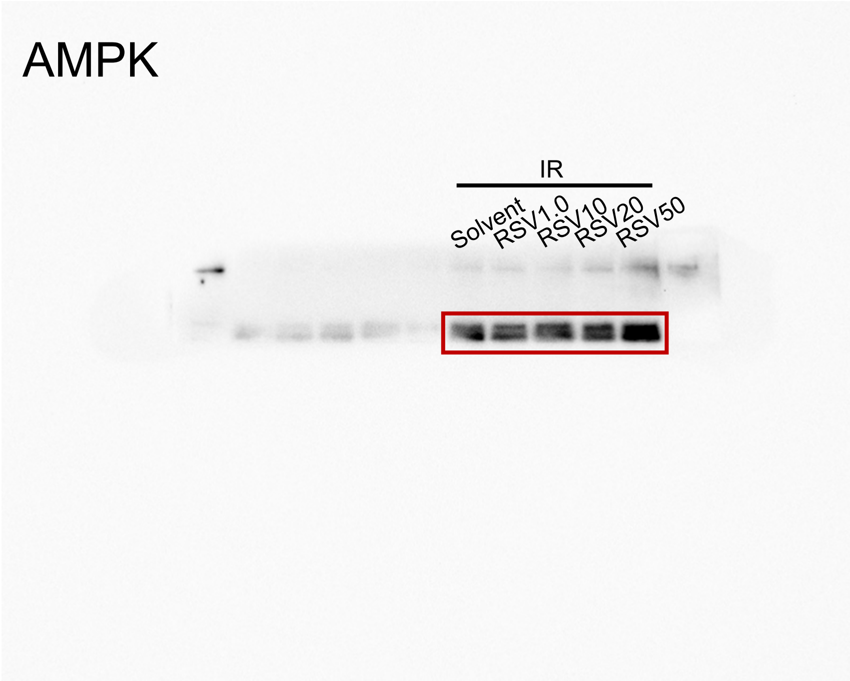

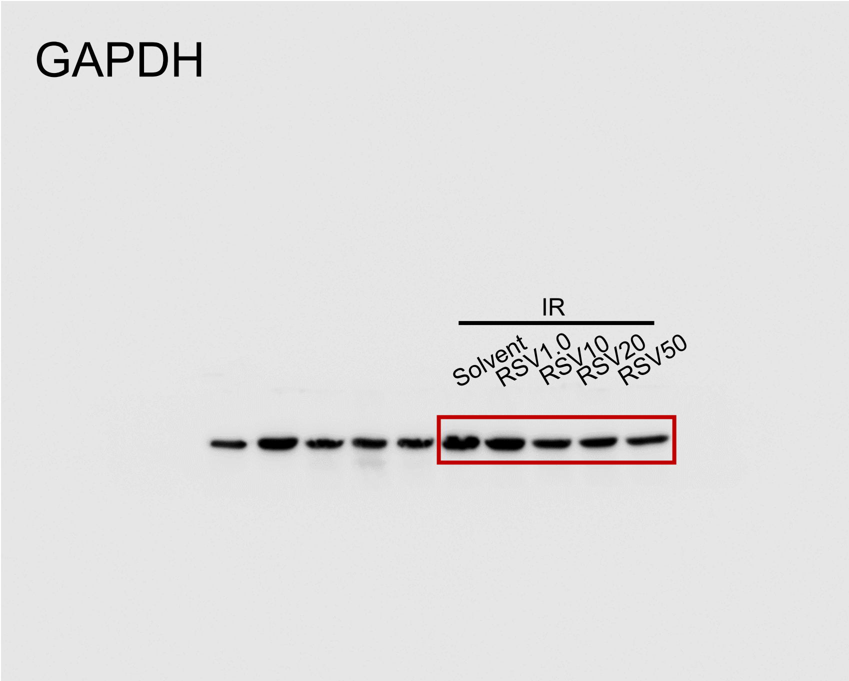

Related to Figure S2b

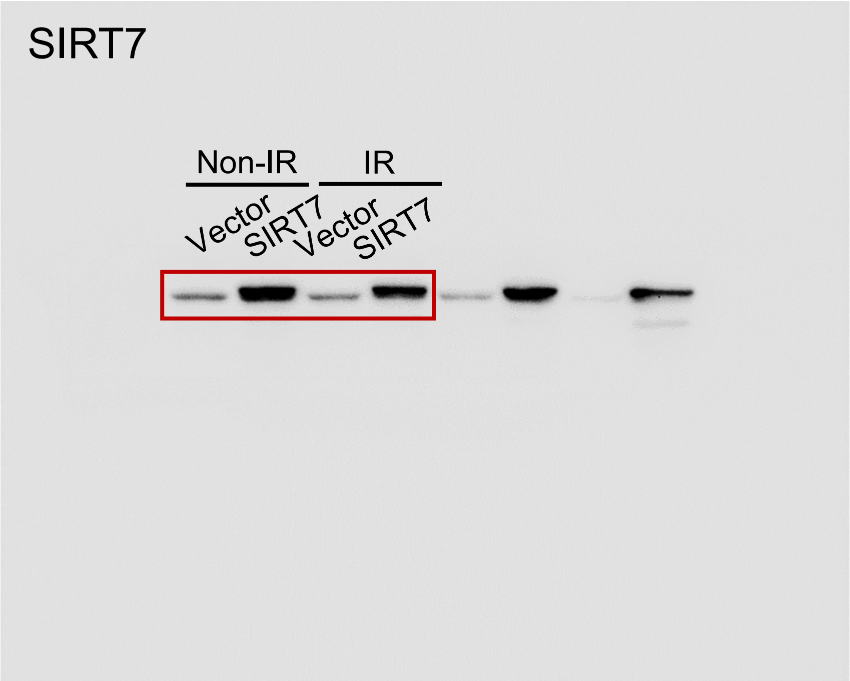

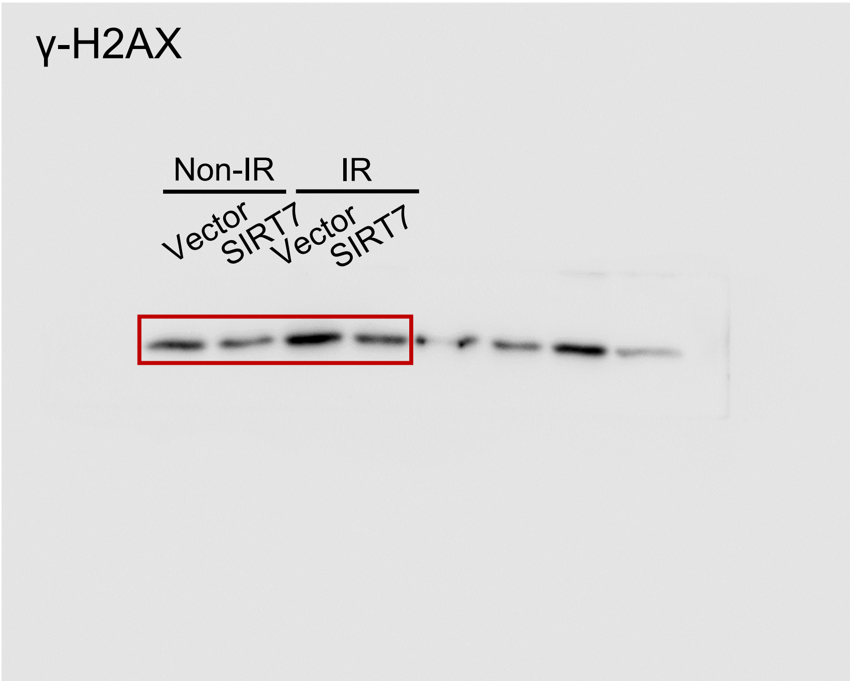

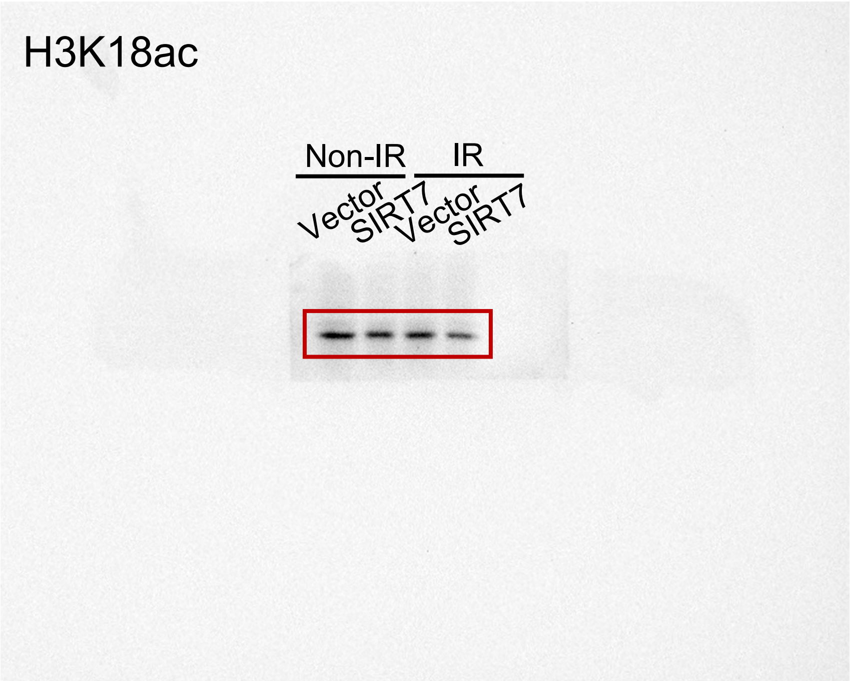

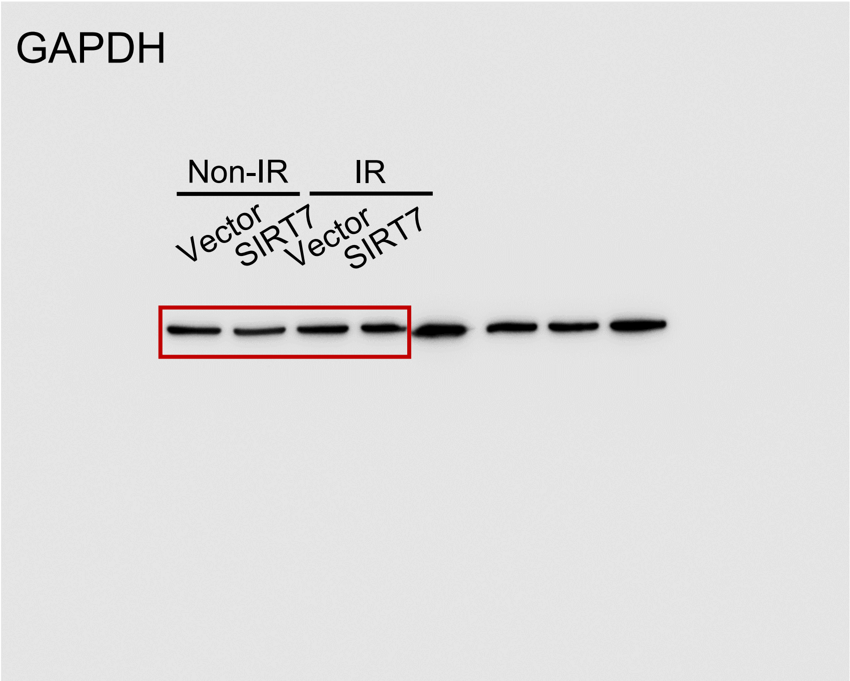

Supplement: Supplementary file 6 — Original Data [file 41419_2022_5281_MOESM6_ESM.pdf]
